# Genomic characterization of bZIP gene family and patterns of gene regulation on *Cercospora beticola Sacc* resistance in sugar beet (*Beta vulgaris* L.)

**DOI:** 10.3389/fgene.2024.1430589

**Published:** 2024-07-30

**Authors:** Xiao Yin, Yu Liu, Yunhe Gong, Guangzhou Ding, Chunlei Zhao, Yanli Li

**Affiliations:** ^1^ College of Modern Agriculture and Ecologcial Environment, Heilongjiang University, Harbin, China; ^2^ Sugar Beet Engineering Research Center of Heilongjiang, Harbin, China

**Keywords:** sugar beet, bZIP gene family, *cercospora* leaf spot (CLS), *Cercospora beticola Sacc* (*C. beticola*), disease resistance

## Abstract

Sugar beet (*Beta vulgaris* L.) is one of the most important sugar crops, accounting for nearly 30% of the world’s annual sugar production. And it is mainly distributed in the northwestern, northern, and northeastern regions of China. However, *Cercospora leaf* spot (CLS) is the most serious and destructive foliar disease during the cultivation of sugar beet. In plants, the bZIP gene family is one of important family of transcription factors that regulate many biological processes, including cell and tissue differentiation, pathogen defense, light response, and abiotic stress signaling. Although the bZIP gene family has been mentioned in previous studies as playing a crucial role in plant defense against diseases, there has been no comprehensive study or functional analysis of the bZIP gene family in sugar beet with respect to biotic stresses. In this study, we performed a genome-wide analysis of bZIP family genes (BvbZIPs) in sugar beet to investigate their phylogenetic relationships, gene structure and chromosomal localization. At the same time, we observed the stomatal and cell ultrastructure of sugar beet leaf surface during the period of infestation by Cercospora beticola Sacc (*C. beticola*). And identified the genes with significant differential expression in the bZIP gene family of sugar beet by qRT-PCR. Finally we determined the concentrations of SA and JA and verified the associated genes by qRT-PCR. The results showed that 48 genes were identified and gene expression analysis indicated that 6 BvbZIPs were significantly differential expressed in *C. beticola* infection. It is speculated that these BvbZIPs are candidate genes for regulating the response of sugar beet to CLS infection. Meanwhile, the observation stomata of sugar beet leaves infected with *C. beticola* revealed that there were also differences in the surface stomata of the leaves at different periods of infection. In addition, we further confirmed that the protein encoded by the SA signaling pathway-related gene BVRB_9g222570 in high-resistant varieties was PR1, which is closely related to systemic acquired resistance. One of the protein interaction modes of JA signal transduction pathway is the response of MYC2 transcription factor caused by JAZ protein degradation, and there is a molecular interaction between JA signal transduction pathway and auxin. Despite previous reports on abiotic stresses in sugar beet, this study provides very useful information for further research on the role of the sugar beet bZIP gene family in sugar beet through experiments. The above research findings can promote the development of sugar beet disease resistance breeding.

## 1 Introduction

Transcription factors (TFs) play regulatory roles in the growth and development of organisms. The functional properties of TFs are essential for understanding the transcriptional regulatory network and the biological processes involved (Liu et al., 2014). The basic leucine zipper (bZIP) TF family is one of the largest transcription factor families (Hou et al., 2022), containing the highly conserved structural domain of bZIP, which is widely found in eukaryotic organisms (Jakoby et al., 2002). Structurally, the highly conserved structural domain of bZIP has two regions, a basic DNA binding region and a leucine-zipper region. This basic region consists of approximately 16 amino acid residues containing a nuclear localization signal and an invariant N-X7-R/K motif in contact with the DNA (Jakoby et al., 2002). This motif binds preferentially to DNA sequences containing core ACGT cis-acting elements such as A-box (TACGTA), C-box (GACGTC) and G-box (CACGTG) (Izawa et al., 1993). On the other hand, the leucine zipper is less conserved with a heptapeptide repeat of leucine or other hydrophobic amino acids and involved in idiosyncratic identification and dimerization (Zhang et al., 2020).

The bZIP TFs play a critical role in regulating many transcriptional responses in multiple biological processes in plants. For instance, they regulate the development of organs and tissues during plant growth and development, including vascular bundles (Yin, 1997), photomorphogenesis (Xiao et al., 2022), embryogenesis (Guan et al., 2009), and seed maturity and germination (Zhao et al., 2020). In addition, bZIP TFs are involved in responses to abiotic and biotic stresses, such as low-temperature stress, water deficit, high salinity and defense against pathogens (Sornaraj et al., 2016; Gong et al., 2022). Along with advances in sequencing technology, more and more plant genomes are being detected. Up to date, the bZIP gene family has been systematically identified in many plants such as quinoa (Li et al., 2020), Arabidopsis (Dröge-Laser et al., 2018), rice (Nijhawan et al., 2007), cotton (Zhang et al., 2022), maize (Wei et al., 2012), potato (Wang et al., 2021), tomato (Li et al., 2015), wheat (Liang et al., 2022), banana (Hu et al., 2016), carrot (Que et al., 2015), cucumber (Mehmet et al., 2014), pomegranate (Wang et al., 2022) etc.

However, a large number of studies on plant bZIP TFs have been related to their involvement in abiotic stresses. In contrast, little research has been done on the biotic stress aspects of bZIP transcription factors. At present, some bZIP genes have been found in many plants for defense against pathogens. Through the salicylic acid signaling pathway, over-expression of Vitis vinifera VvbZIP60 enhances Arabidopsis resistance to powdery milde (Yu et al., 2019). FabZIP46 played an active role in the protection of strawberries against *Botrytis cinerea*. Moreover, over expression of FabZIP46 significantly delayed the onset of *B. cinerea* in strawberries and reduced morbidity rates (Lu et al., 2020). The LrbZIP1 was isolated from lily plants and was found to significantly inhibit the growth of Fusarium oxysporum by over-expression in tobacco (Zhang et al., 2014). RcbZIP17 is associated with resistance to gray mold in roses based on virus-induced gene silencing (VIGS) and overexpression (OE) studies (Li et al., 2023). TabZIP1 gene isolated from wheat may be an ET/MeJA-dependent signal transduction pathway involved in resistance and defense response to infection by stripe rust pathogen (Zhang et al., 2008). The OsbZIP1 gene, which was identified from rice, was found to enhance rice defense against rice *Magnaporthe grisea* possibly through ABA, SA, and JA signaling transduction pathways (Meng et al., 2005). In addition, the bZIP transcription factors in maize also exhibit very prominent functions in response to pathogen attack, such as their increased expression during infection of maize with *Ustilago maydis* (Wei et al., 2012). The above studies have shown that bZIP genes can be used individually or synergistically to defend against pathogens and regulate plant growth and development in complex external environments.

Sugar beet (*Beta vulgaris *L.) belongs to the *Amaranthaceae* family (formerly the *Chenopodiaceae*) and is one of the world’s most important sugar crops and one of the main nutrients (Porcel et al., 2018). And sugar beet is ranked second in sugar production after Sugarcane (*Saccharum officinarum L.*). Although improved breeding and cultivation techniques have increased sugar beet yields and sugar production in recent decades, many abiotic and biotic stresses continue to exist, which affects sugar beet growth and sugar production. *Cercospora* leaf spot (CLS) is the most serious and destructive foliar disease of sugar beet (Heitefuss, 2010). This disease is caused by the airborne fungus *Cercospora beticola Sacc* (*C. beticola*). And CLS is most pernicious in warm, humid growing regions (Heitefuss, 2010). A key option for the integrated management of CLS on sugar beet is to cultivate resistant varieties. The improvement of CLS resistance in sugar beet varieties has been a joint effort of geneticists and breeders over the past decades. Wild sea beet (B. *vulgaris subsp. Maritima*) has been a source of CLS resistance genes for a long time; And precise mapping of resistance QTLs was used to help marker-assisted selection (MAS) in breeding programs to introgress CLS resistance. The research result showed that CLS resistance is typically managed by at least four identifiable quantitative trait loci (QTLs), and the more precise the mapping, the higher the chances of breaking the potential linkage between CLS resistance (Rangel et al., 2020). Interestingly, the underlying gene products can also be identified and used as molecular markers to identify alleles associated with resistance. Integration of molecular markers in conventional breeding procedures has provided a reliable means for improving the efficiency of selection methods (Norouzi et al., 2017). The more important is how to find out the molecular markers. Although few studies have focused on the molecular basis of defense in sugar beet, there is some published research examining plant defenses upon *C. beticola* infection. The interaction between sugar beet and *C. beticola* begins with an initial defence response by the plant up-regulating phenylalanine ammonia-lyase (PAL) involved in the various biosynthetic pathways for many plant-related SM compounds (Schmidt et al., 2008). ABA was also found to reduce PAL gene expression in sugar beet through an unknown mechanism. Genes were activated in hormone production (ethylene, jasmonic acid, and gibberellin), lignin and alkaloid synthesis, signalling, and pathogenesis-related (PR) genes by the time symptoms had appeared (Schmidt et al., 2008).

The classification of varietal reaction to CLS is based on symptomatic leaf area (susceptibility) and the resulting relative yield loss (tolerance), so decrease symptomatic leaf area, to reduce the yield loss is one of the breeding objectives (Vogel et al., 2018). Monogenic resistant genotype are different with partial resistance genotype in that although they all had a stronger defense response than susceptible genotype, the pathogen can still infect partially resistant genotyped varieties and cause disease (Vogel et al., 2018). In present, CRISPR/Cas 9 technology have been applied to major sugar beet diseases for pathogen (bacterial/fungal/viral) resistance. For CLS, many potential tolerance/resistance genes have been used such as SP1 and SP2 (Acid chitinase activity), SE1 (Chitinase activity), SE2 (Exochitinase activity), qcr1 and qcr4(QTL disease resistance) (Misra et al., 2023).

The defense functions of many members of the bZIP gene family have been extensively studied, especially in Arabidopsis (Jakoby et al., 2002). Although the sugar beet bZIP gene family was identified in abiotic stresses (Gong et al., 2022), no bZIP genes have been identified regarding the involvement of sugar beet in disease resistance. Therefore, we conducted a genome-wide analysis of the bZIP gene family in the hopes of providing a theoretical foundation for further research into the biological functions of the bZIP gene family in sugar beet.

## 2 Materials and methods

### 2.1 Identification of bZIP family in sugar beet

The sugar beet (*B. vulgaris L.*) genome database was downloaded from Ensembl Plants (https://plants.ensembl.org, accessed on 16 April 2023). The Arabidopsis bZIP sequences were derived from the Arabidopsis Information Resource (TAIR) (http://www.arabidopsis.org, accessed on 26 March 2023) (Dröge-Laser et al., 2018). We downloaded the Hidden Markov Model profiles of bZIP domains (PF00170 and PF07716) from Pfam (http://pfam.xfam.org/, accessed on 16 April 2023), and searched in sugar beet representative protein sequences by using HMMER software (Johnson et al., 2010). Finally, all output candidate genes were verified by using the Conserved Domain Database (Lu et al., 2019) and SMART (https://smart.embl.de/, accessed on 16 April 2023) recognizes BvbZIP proteins.

### 2.2 Physicochemical properties and subcellular localization analysis of BvbZIPs

The physicochemical properties of the predicted BvbZIPs were analyzed by using the ProtParam online tool (https://www.expasy.org/, accessed on 24 April 2023), including theoretical isoelectric point (pI), molecular weight (MW) and hydrophilicity index (Wilkins et al., 2019). Subcellular localizations of the BvbZIP genes were predicted by the online tool Cell-PLoc 2.0 (Chou and Shen, 2008). The results of BvbZIP genes analysis are shown in Table 1.

**TABLE 1:** Features of sugar beet bZIP transcription factors.

		Chromosome	Start	End	Protein (aa)	MW (Da)	PI	Instability index	Aliphatic index	GRAVY	Subcellular localization
BvbZIP1	BVRB_1g001330	1	1455782	1459379	347	39115.74	8.99	67.88	61.64	−0.882	Nucleus
BvbZIP2	BVRB_1g003640	1	3977905	3982930	420	44611.56	6.16	46.48	68.33	−0.696	Nucleus
BvbZIP3	BVRB_1g005070	1	5536427	5546380	532	58415.38	6.27	64.72	59.94	−0.873	Nucleus
BvbZIP4	BVRB_1g006910	1	7653025	7654107	157	18101.21	5.69	48.99	76.37	−0.782	Nucleus
BvbZIP5	BVRB_1g011350	1	18474761	18486025	361	39296.47	8.03	61.54	64.68	−0.759	Nucleus
BvbZIP6	BVRB_1g011670	1	19888068	19896052	270	29494.53	5.55	57.94	71.59	−0.607	Nucleus
BvbZIP7	BVRB_1g013750	1	29718550	29722391	305	34711.21	4.78	51.32	79.90	−0.621	Nucleus
BvbZIP8	BVRB_1g021380	Bvchr1_un.sca006	501467	512486	360	40871.49	6.78	51.94	83.86	−0.411	Nucleus
BvbZIP9	BVRB_2g025990	2	2777445	2781679	269	29655.86	6.06	35.81	69.59	−0.700	Nucleus
BvbZIP10	BVRB_2g028580	2	5556253	5557110	184	21454.78	5.25	72.33	73.64	−0.925	Nucleus
BvbZIP11	BVRB_2g035120	2	15713695	15722570	346	37853.6	5.95	50.51	63.18	−0.743	Nucleus
BvbZIP12	BVRB_2g041830	2	36849536	36858434	455	50104.92	6.16	56.23	80.90	−0.526	Nucleus
BvbZIP13	BVRB_2g042510	2	37909460	37913344	406	45708.5	5.36	70.34	67.56	−0.647	Nucleus
BvbZIP14	BVRB_2g042860	2	38412982	38417069	168	18153.98	9.90	62.46	58.81	−1.102	Nucleus
BvbZIP15	BVRB_3g050990	3	3093968	3095272	173	19532.92	6.13	63.45	67.63	−0.670	Nucleus
BvbZIP16	BVRB_3g052640	3	4903706	4911000	357	38172.38	6.27	58.70	57.70	−0.737	Nucleus
BvbZIP17	BVRB_3g053880	3	6266761	6267695	169	18891.22	8.81	64.38	67.46	−0.805	Nucleus
BvbZIP18	BVRB_3g057700	3	12517970	12529096	388	42862.62	6.02	55.57	63.99	−0.813	Nucleus
BvbZIP19	BVRB_3g060650	3	19135854	19150427	577	63771.86	7.17	68.17	64.94	−0.765	Nucleus
BvbZIP20	BVRB_3g062960	3	23089361	23097205	449	49511.63	7.12	54.20	82.23	−0.472	Nucleus
BvbZIP21	BVRB_3g063410	3	23723506	23726800	690	75235.28	5.66	70.71	51.50	−0.536	Nucleus
BvbZIP22	BVRB_3g064480	3	25013662	25018361	333	36540.82	9.27	49.24	67.60	−0.807	Nucleus
BvbZIP23	BVRB_3g070510	Bvchr3_un.sca012	459941	463981	164	19014.36	5.47	59.74	76.65	−1.068	Nucleus
BvbZIP24	BVRB_4g081830	4	12442145	12448104	435	47155.21	6.47	56.14	63.91	−0.755	Nucleus
BvbZIP25	BVRB_4g086160	4	24868899	24873829	353	38594.4	5.73	65.96	57.51	−0.929	Nucleus
BvbZIP26	BVRB_4g094850	Bvchr4_un.sca014	1102729	1103880	275	30803.62	5.03	37.92	63.53	−1.024	Nucleus
BvbZIP27	BVRB_5g107170	5	16232104	16244893	397	42142.54	5.85	66.99	51.79	−0.808	Nucleus
BvbZIP28	BVRB_5g121770	5	51617727	51618936	141	16210.4	5.87	58.62	75.32	−0.763	Nucleus
BvbZIP29	BVRB_6g128410	6	1715145	1716294	289	32878.42	8.49	60.09	65.50	−0.948	Nucleus
BvbZIP30	BVRB_6g129360	6	2809863	2811181	180	20240.99	5.36	54.12	53.17	−0.937	Nucleus
BvbZIP31	BVRB_6g134230	6	8140667	8148087	240	26691.85	9.35	75.60	56.96	−0.697	Nucleus
BvbZIP32	BVRB_6g135660	6	10485479	10490603	363	41231.75	6.12	56.10	83.86	−0.463	Nucleus
BvbZIP33	BVRB_6g140280	6	20681612	20682470	169	20087.54	6.08	59.81	78.46	−0.876	Nucleus
BvbZIP34	BVRB_6g140290	6	20809397	20810258	165	19236.24	5.82	70.12	76.30	−0.920	Nucleus
BvbZIP35	BVRB_7g159570	7	3838771	3848492	451	49190.8	8.30	62.76	58.69	−0.793	Nucleus
BvbZIP36	BVRB_7g159880	7	4346188	4351319	465	51639.47	6.87	65.85	63.55	−0.807	Nucleus
BvbZIP37	BVRB_7g169340	7	35447574	35451886	489	51591.33	9.49	47.09	62.07	−0.655	Nucleus
BvbZIP38	BVRB_7g175790	7	43158350	43161974	386	42515.86	5.69	57.22	57.69	−0.836	Nucleus
BvbZIP39	BVRB_7g176150	7	43480562	43492442	514	57287.99	7.84	61.34	68.44	−0.682	Nucleus
BvbZIP40	BVRB_8g186270	8	8997653	8998577	208	23844.94	5.76	81.62	87.69	−0.717	Nucleus
BvbZIP41	BVRB_9g207080	9	11871415	11882108	264	29459.13	6.92	51.19	74.92	−0.653	Nucleus
BvbZIP42	BVRB_9g209240	9	21452871	21456705	349	39180.09	5.67	64.28	60.06	−1.019	Nucleus
BvbZIP43	BVRB_9g214060	9	33644472	33649226	290	31705.93	5.89	39.42	58.24	−0.838	Nucleus
BvbZIP44	BVRB_9g221110	9	41736484	41743228	380	42463.07	8.65	63.22	64.26	−0.915	Nucleus
BvbZIP45	BVRB_9g224390	Bvchr9_un.sca001	13176	16070	572	62254.67	6.94	55.35	57.20	−0.905	Nucleus
BvbZIP46	BVRB_005360	0139.scaffold00419	5587	16531	194	21854.28	6.60	59.35	59.43	−0.923	Nucleus
BvbZIP47	BVRB_011410	0316.scaffold00796	83146	94374	353	37059.41	5.23	51.89	47.68	−0.860	Nucleus
BvbZIP48	BVRB_012640	0390.scaffold00899	52259	61411	434	48475.43	6.06	44.12	80.97	−0.458	Nucleus

### 2.3 Multiple sequence alignment and analysis of phylogenetic tree

The entire bZIP protein sequences of the two plants species (sugar beet and the model plant species Arabidopsis) were aligned with the MUSCLE program by using the default parameters in the online tool Clustal Omega (https://www.ebi.ac.uk/Tools/msa/clustalo/, accessed on 24 April 2023) (Sievers and Higgins, 2017). The obtained phylogenetic tree was visualized by the online iTOL (Interactive Tree of Life) (https://itol.embl.de/, accessed on 24 April 2023) All isolated and characterized BvbZIP proteins were classified into subfamilies according to Arabidopsis (Bailey et al., 2015).

### 2.4 Analyses of gene structure, domain and conserved motif compositions of BvbZIPs

Distribution patterns of exons and introns of each BvbZIP genes were found through general feature format (GFF3) files and visualized by using TBtools software (Chen et al., 2020). The conserved domains were defined by using the Batch-CD search (https://www.ncbi.nlm.nih.gov/Structure/bwrpsb/bwrpsb.cgi, accessed on 25 April 2023) (Lu et al., 2019) with default parameters. The MEME analyzing tool were used to identify the motifs in all BvbZIP proteins with maximum number of 12 motifs and all other parameters set to default (Bailey et al., 2015). The figure was represented by using TBtools software (Chen et al., 2020).

### 2.5 Chromosomal distribution and collinear analysis with other species

Gene positions of BvbZIPs on the chromosomes were gained from the GFF3 file in Ensembl Plants (https://plants.ensembl.org, accessed on 20 April 2023) and visualized through TBtools software (Guan et al., 2022). All BvbZIPs were localized to sugar beet chromosomes by using Circos based on the physical location information in the sugar beet genome database (Krzywinski et al., 2009). We used the default parameters of the Advance Circos package in the TBtools to detect gene duplication. Homology between bZIPs in sugar beet and the other 4 species (*Arabidopsis thaliana*, *Glycine max*, *Medicago truncatula* and *Zea mays*) was analysed through TBtools software.

### 2.6 Cis-element analyses of BvbZIP genes

To predict the composition of cis-acting elements, we submitted the upstream 2-kb sequence of each sugar beet bZIP from the initiation codon ATG to the PlantCARE tool (http://bioinformatics.psb.ugent.be/webtools/plantcare/html/, accessed on 18 May 2023) (Lescot et al., 2002).

### 2.7 Predicted interacting protein network

STRING database (https://string-db.org/, accessed on 22 May 2023) was used to analyze molecular interaction networks of BvbZIP proteins.

### 2.8 Stomatal observation and cell ultrastructure observation of sugar beet leaf surface

After 15 days of infection, the infection of the stomata of sugar beet leaves was observed under an optical microscope. Sugar beet leaves have two varieties of disease-resistant and susceptible. In this experiment, the leaves within 15 days after infection were sampled every day, and the healthy leaves were used as the control. The samples were prepared by sampling, fixing, pre-washing, post-fixing, rinsing, dehydration, infiltration, polymerization, sectioning and staining. Finally, the fixed sections were observed and photographed by JEM-1230 transmission electron microscope.

### 2.9 Analysis of the potential role of BvbZIP gene family in CLS resistance

#### 2.9.1 Infection experiment

We used two sugar beet varieties, KWS5145 (highly susceptible to CLS) and F85621 (highly resistant to CLS). And these two varieties were subjected to separate experimental treatments, divided into the control group, the early infection test group and the disease test group. When the seedlings were three pairs of true leaves, the *C. beticola* was introduced by the spray method. Both the initial infection test group and the onset test group were uniformly sprayed with the same concentration of spore suspension, while the control group was uniformly sprayed with the same amount of sterilized water. These treated plants were cultivated in an environment of light culture with 90% humidity and 26°C(high temperature and high humidity) and were sampled for the next step of the experiment according to the previous experimental design.

#### 2.9.2 Sampling treatment and data processing of transcriptome

When the test material was in three pairs of true leaves, 9 plants of each of KWS5145 highly susceptible variety and F85621 highly resistant variety with good growth were taken, and each variety was divided into three groups with three replicates, which were the control group, the test group at the early stage of infestation and the test group at the onset of disease. The three samples of the high-susceptible variety KWS5145 control group were numbered HS_CK_1, HS_CK_2, HS_CK_3, and the three samples of the high-resistant variety F85621 control group were numbered HR_CK_1, HR_CK_2, HR_CK_3. The infection test group were numbered HS_infected_1, HS_infected_2, HS_infected-3, and the three samples of the high-resistant variety F85621 control group were numbered HR_infected_1, HR_infected_2, HR_infected_3. The three samples of the high-susceptible variety KWS5145 in the experimental group were numbered HS_disease_1, HS_disease_2, HS_disease_3, and the three samples of the high-resistant variety F85621 in the experimental group were numbered HR_disease_1, HR_disease_2, HR_disease_3. The operating table was air-purified and sterilized, and the leaves were washed with sterile water and left to air dry. The spore suspension was sprayed evenly on the surface of the leaves of the two varieties of the early infestation test group and the onset test group, and placed in a light culture room at 26°C and 80% relative humidity in isolation; the control group of the two varieties was sprayed on the surface of the leaves with an equal amount of distilled water and placed in a separate space at 26°C and 80% relative humidity for cultivation. These samples taken above were stored at −80°C.

Transcriptome sequencing: A total of 18 samples from 6 experimental groups were sent to Shanghai Major Biomedical Technology Co. for RNA extraction and transcriptome sequencing.

Transcriptome data quality analysis: The Illumina platform converted the sequencing image signal into text by CASAVA base identification and stores it in fastq format as raw data. The quality assessment of raw sequencing data for each sample is performed with Fastp software, including base quality distribution statistics, base error rate distribution statistics, and base content distribution statistics. The SeqPrep and Sickle software were used to quality control the raw data in terms of splice sequences, low quality read segments, uncertain base rates, and too short lengths, to obtain high-quality data, and the quality controlled data were again counted and evaluated in the same way as the raw data.

#### 2.9.3 RNA extraction and quantitative real-time PCR (*q*RT-PCR) analysis

The collected samples were quickly ground into powder form using liquid nitrogen. The ground 100 mg of powder was transferred to a 1.5 mL centrifuge tube for total RNA extraction. Total RNA was isolated and purified according to Kobayashi et al. (2010). In addition, RNA was reverse transcribed into cDNA using this PrimeScript™ RT reagent Kit with gDNA Eraser (Perfect Real Time) (Takara, Dalian, China) according to the manufacturer’s instructions. A three-step PCR amplification reaction was also performed using cDNA as a template. After the amplification cycle, it was cooled to 60°C, and then heated to 95°C to denature the DNA product. The specific primers used for real-time fluorescence quantitative PCR analysis are shown in Table 2. In performing real-time fluorescence quantitative PCR, gene expression is typically measured in three independent biological replicates, each using at least two technical replicates.

**TABLE 2:** PCR primers used in the research.

Primer name	Forward primer (5ʹ-3ʹ)	Reverse primer (5ʹ-3ʹ)
BvbZIP1	GAA​CCA​ATG​TGA​TAC​TAC​TG	CCAACTGTGCTGATACTT
BvbZIP2	TCC​TCT​TCA​CCT​TCT​TCT​CT	CCA​TAA​TCA​CTC​AGC​AAC​AC
BvbZIP3	CAGCATCAGCAGCATCA	GGAGCCTCAGCATCTTG
BvbZIP4	GTC​CTG​TGT​CCT​GCT​ACT​G	CCT​CTC​ATC​CAT​TGT​TGT​TGT
BvbZIP5	GGC​GAC​GAA​GAC​GAT​GAT​T	GAG​GAG​AAG​GAG​AAG​GAG​GAA
BvbZIP6	TCA​TCG​TCA​TCA​TCG​TCA​TCA	TCT​CAG​CAT​CAA​GCA​AGG​TAT
BvbZIP7	AAG​AAG​CAG​GAG​TAG​GAG​TAG	GCCACGGAAGCACCATA
BvbZIP8	AAT​CGG​CAG​AAT​AGT​GAA​C	CGG​AAG​AGG​TGA​TAA​TAG​TG
BvbZIP9	GCA​TAC​CGC​CTA​CTT​AGA​AG	CGA​CCA​TTA​CCA​ACA​CTC​AA
BvbZIP10	ATT​CAC​TCC​ACA​TCC​ATC​AT	TAT​CAT​CCT​TCT​CTG​CTT​CC
BvbZIP11	AAG​CAG​GAG​ACA​ATC​AAG​GAA	TAGAACGGCGAGCAGACT
BvbZIP12	GGCTCATCAGGCTCATC	TTG​TTG​GCG​TAA​GTT​ATC​AG
BvbZIP13	GTA​AGC​CAA​GAA​TGT​CGC​AAT	GGT​AGA​TGT​CCA​AGA​GGT​GAG
BvbZIP14	ATCTCACGGCTCCTCTCA	TCA​TCA​TCA​CTC​TCC​ATT​CCT
BvbZIP15	GAGTTCGGAGGAGGAGAT	CTGCTGTGAGGTGATGTT
BvbZIP16	CTT​CTG​TTC​CTA​TGT​GTA​TGC	ATTGCCGTTGCCTGATG
BvbZIP17	AGTGTTGCTCTTCCTAC	TCTTCTCGCTGATTCTC
BvbZIP18	TTC​AAG​AAC​GCC​AAC​TAC​A	CCG​AGA​TAC​AAC​GAC​TAC​AA
BvbZIP19	TCA​TCA​TCA​GCA​TCA​GCA​TCA	CGA​GGA​CGA​CGA​AGA​GTT​G
BvbZIP20	CTT​GGC​TTC​TGT​GTC​TGT​TG	TAT​CCT​CGG​TAT​CGT​CTG​TTG
BvbZIP21	GAT​GGT​GAA​GGC​GTG​ATT​AGA	ACT​GTG​GTG​GAG​ATG​GAT​ACT
BvbZIP22	CTT​CCA​CAG​CAG​CAA​CAA​CA	GCC​TCC​TCT​CAA​CAG​TCT​TCT
BvbZIP23	TTG​CCT​CTT​CTT​CCT​ACT​ACT	TCA​CTA​CCA​CCA​CCA​TTG​T
BvbZIP24	CAT​CAT​CAT​CAT​ACG​ACT​AC	GAATCAACGAGCACACT
BvbZIP25	AGA​GTG​AGC​CTG​GTG​AAG​T	TTGTGCCGATTGCCGATT
BvbZIP26	AAT​CAT​CAG​TTC​ACC​ATC​CA	GCT​ACC​GTT​GTT​ATT​GTT​GT
BvbZIP27	CCGCTTCGTCTCCTATC	GCCAACTCATCGCATTC
BvbZIP28	CCAGATGCGGAGGATTC	GTG​TTG​TTG​CTT​CTT​CAT​TC
BvbZIP29	GGA​AGC​GTA​AGC​GAA​TGA​A	TAA​GCC​GTA​AGT​GGT​TGG​A
BvbZIP30	GAACACGAACACGAACAC	CTG​AGG​TAG​ACA​CAA​TAG​GAT
BvbZIP31	CAA​GTG​GAG​CAG​GAG​ATT​CG	AGT​AGG​AGG​AGG​AGG​AGG​AG
BvbZIP32	GCC​TCT​CCA​TCA​GAT​TAG​T	CGCCACCAACATACATAC
BvbZIP33	AGGATGCGGAAGAAGAG	CTG​ACG​AAG​GTT​AGA​AGT​T
BvbZIP34	TCT​TAC​ACC​TCC​ATT​ACC​A	ATT​CCT​GCT​AAC​TCA​CTT​G
BvbZIP35	GCG​AGG​AGC​AGA​ACA​TAC​A	AGA​CAA​CAG​AAG​AGC​ATC​ACT
BvbZIP36	CTGGTCCTGTTCGCAAT	CAT​AAT​AAG​CCT​GTC​TCC​TAC
BvbZIP37	GCC​GAG​GTG​TTG​AGT​TGA​TG	TGT​TGC​TGC​TGC​TGT​TGT​G
BvbZIP38	AAC​TGT​AGA​CTG​GAG​AAG​AAC	CAT​TGG​AAG​AAG​GTG​AAG​GT
BvbZIP39	CGACAAGCAGCAAGATG	TAGGACGAGACGACCAA
BvbZIP40	ACT​CTT​ATC​TTC​CAC​TCC​AT	TCA​TTC​TCT​TCA​GCC​TTC​TA
BvbZIP41	AATCTGCTGCTCGTTCTC	CCT​TAT​GTT​CTG​CTC​CTC​AA
BvbZIP42	CAA​GAA​GCA​ATG​AAG​CGA​GAA	ATG​ATG​ATG​AAG​GTG​GTG​GAA
BvbZIP43	GGCTTGCTTGGCTATGTC	TGT​TGT​TGC​TGT​GAT​TCT​GA
BvbZIP44	TAGCCACAGACGGACAA	GCAGCAGATACGACAGT
BvbZIP45	TCA​TCT​CCA​CCA​CCA​ACA​TC	GTC​ATC​AAC​CAC​TTC​ACC​TTC
BvbZIP46	GCT​ACT​GTT​GCT​GCT​TCT​TC	GCT​TAT​GCC​TCG​GAT​TAC​CA
BvbZIP47	CTCCTGGTGGCGTGTAT	ATC​CTG​TTG​GTT​GTT​GTC​AT
BvbZIP48	AGCAATCCTCTCAACAAG	CCATAGCAATCGCCATT
BvActin	ACT​GGT​ATT​GTG​CTT​GAC​TC	ATG​AGA​TAA​TCA​GTG​AGA​TC

#### 2.9.4 Determination of SA and JA concentration

Using MM-33722O1 plant salicylic acid (SA) ELISA kit and MM-33713O1 plant jasmonic acid (JA) ELISA kit, the concentration of SA and JA in sugar beet leaves were determined by double antibody sandwich method. The samples measured were the control group of high-resistance and high-sensitivity varieties, the experimental group at the early stage of infection and the experimental group at the stage of disease. Each group of samples contained three biological replicates, and each sample was repeated three times. The blank control group, the standard group and the sample group were set up in the measurement process. A standard curve was made using the concentration of the standard as the ordinate and the OD value as the abscissa, replacing the OD value of the sample. The concentrations of SA and JA were calculated respectively. The genes related to its reaction were verified by *q*RT-PCR.

## 3 Results

### 3.1 Genome-wide identification and phylogenetic analysis of BvbZIP genes

**FIGURE 1:**
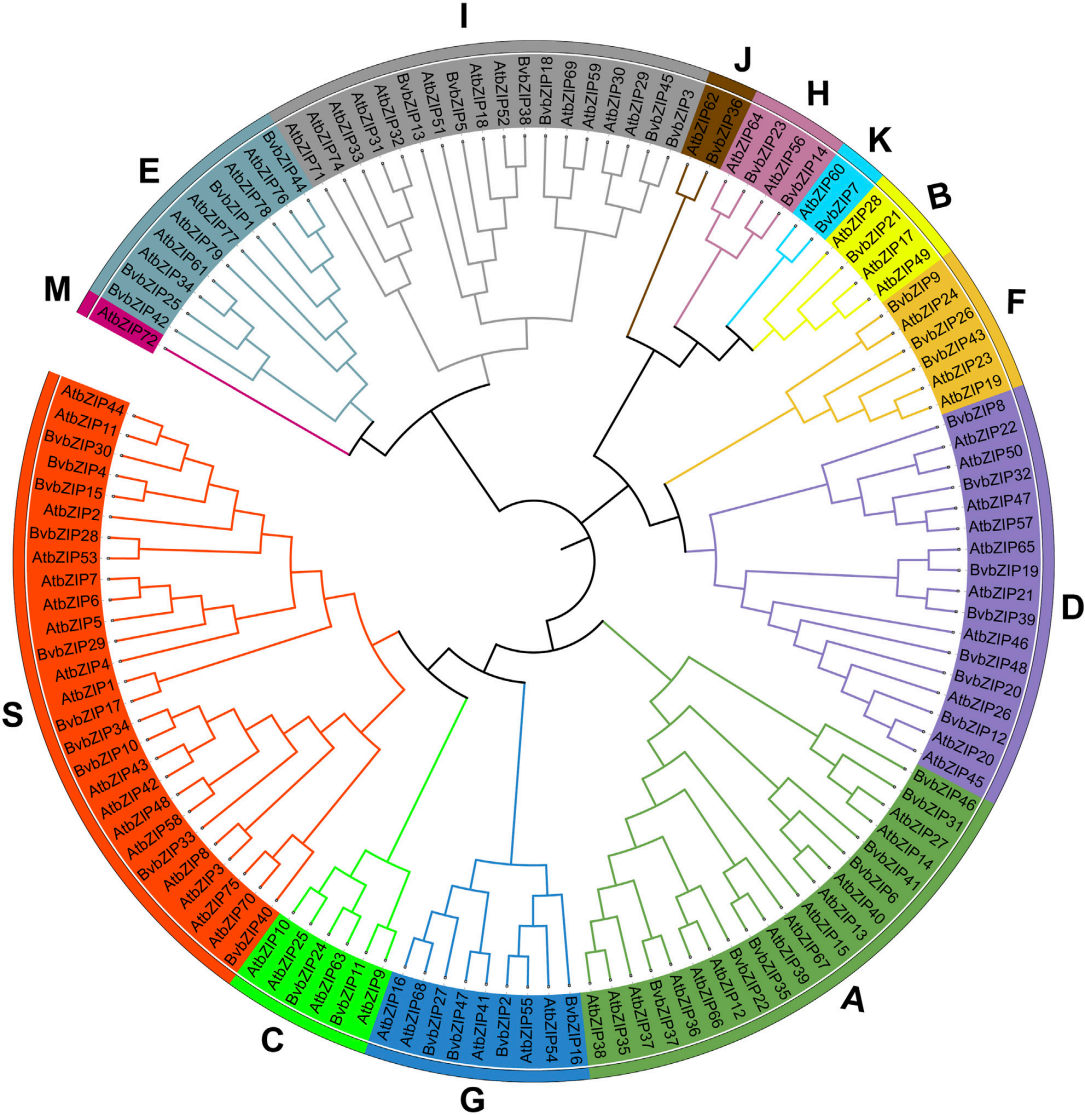
Phylogenetic tree of the bZIP gene family in sugar beet and Arabidopsis. Subfamilies were marked by different colors.

**FIGURE 2:**
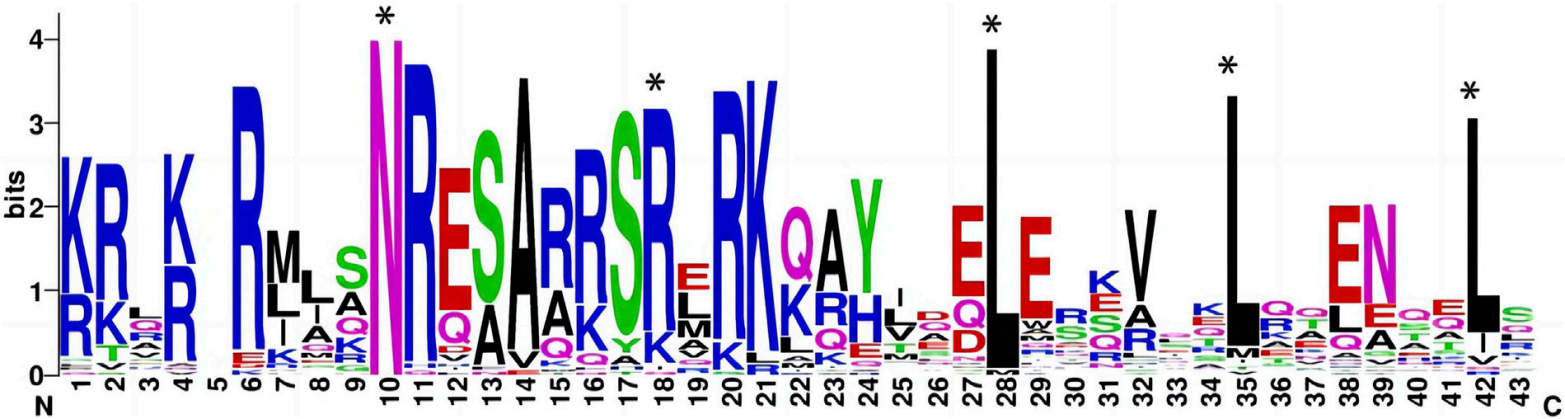
Seqlogo of conserved motif in sugar beet bZIP proteins (The typical sugar beet bZIP structural domain characterization, amino acid sites with highly conserved amino acids are denoted by asterisks).

Based on homology analysis, 48 bZIP genes were identified from the sugar beet genome (Table 1), and the bZIP genes of sugar beet were named according to their chromosomal positions, from BvbZIP1 to BvbZIP48. To assess the evolutionary relationships of the 48 BvbZIP genes, we performed phylogenetic analyses and constructed phylogenetic trees for sugar beet and Arabidopsis based on their the amino acid sequences. Applying the NJ method, we classified proteins into 13 subfamilies: A, B, C, D, E, F, G, H, I, J, K, M, and S (Figure 1). Here, we didn’t identify M subfamily in sugar beet, and it only contains AtbZIP72 (Figure 1). After reclassification, among the BvbZIP subfamilies, S is the largest subfamilies with 10 members, including S1 has 6 BvbZIPs, and S2 has 4 BvbZIPs. A and D subfamily (7), I subfamily (6), E and G subfamily (4), F subfamily (3), C and H subfamily (2) and B, J, K subfamily (1) (Figure 1). Cell-PLoc 2.0 predicted that all BvbZIPs were localized in the nucleus (Table 1). Meanwhile, the core conserved structural domains of BvbZIPs, including the N-X7-R/K basic structural domain and the leucine zipper region, were visualized by multiple sequence comparison of amino acid sequences (Figure 2). And in the typical sugar beet bZIP structural domain characterization, amino acid sites with highly conserved amino acids are denoted by asterisks.

### 3.2 bZIP gene family may be associated with distinct functions

**FIGURE 3:**
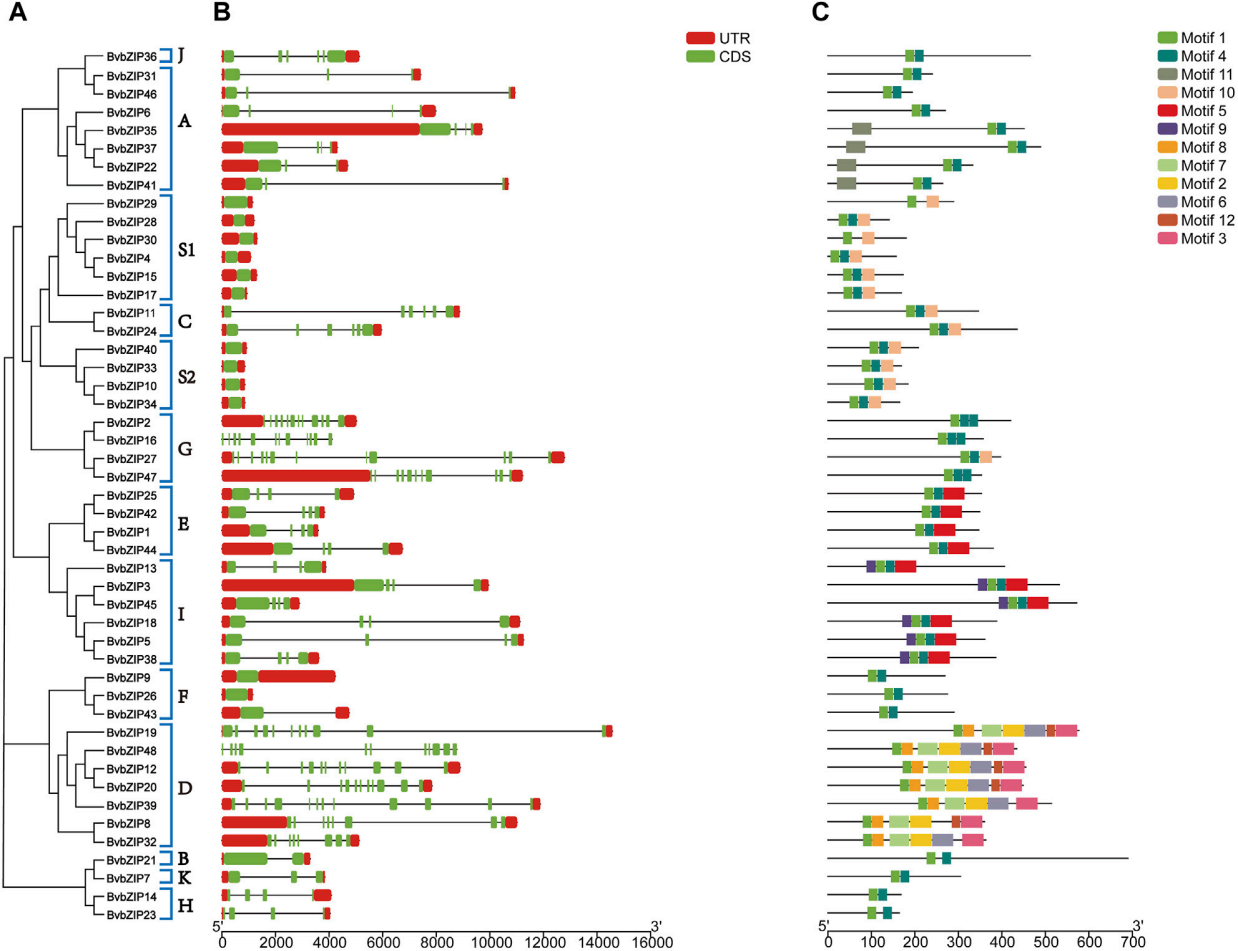
Phylogenetic relationships, gene structure and conserved motifs of the BvbZIP gene family. **(A)** Phylogenetic tree of 48 BvbZIP genes. **(B)** Exon-intron organization of BvbZIP genes. **(C)** Conserved motif distributions of BvbZIP genes.

Conserved motif analysis of the protein sequences of the sugar beet bZIP gene family was performed using the MEME server, and a total of 12 conserved motifs were predicted and visualized by TBtools software (Figure 3C). It can be seen that the distribution and composition of the proportion of conserved motifs is similar among the subfamilies, supporting the results of the phylogenetic tree. Motif 1 existed in every sugar beet bZIP gene. The motif 1 sequence alignment in Pfam indicated that it was the typical bZIP domain. Thus, we infer that the bZIP structural domain is highly conserved, whereas other regions of the bZIP transcription factor are variable. However, the number of motifs varies between subfamilies, for example, subfamily D has the highest number of conserved motifs, with approximately 7 conserved motifs. Since these proteins conserve different motifs in the same subfamily, this reflects the fact that they have different functions (Table 3).

**TABLE 3:** Conserved motif sequences of sugar beet bZIP proteins.

Name	Start	*p*-value	Sites		
BvbZIP31	174	1.59E-23	DEDASGDRRF	KRMIKNRESAARSRARKQAYI	SELELEVVQL
BvbZIP22	266	8.62E-23	VVEKTVERRQ	KRMIKNRESAARSRARKQAYT	HELENKVSRL
BvbZIP37	415	1.82E-22	AVDKVVERRQ	RRMIKNRESAARSRARKQAYT	MELEQEVQKL
BvbZIP35	368	1.82E-22	PVEKVVERRQ	RRMIKNRESAARSRARKQAYT	VELEAELNQL
BvbZIP34	51	5.14E-22	QIQIIDERRQ	RRMISNRESARRSRMRKQRHL	DELWSQVIRL
BvbZIP10	85	5.14E-22	QLTIINERKQ	RRMISNRESARRSRMRKQRHL	DELWSQVVWL
BvbZIP15	36	1.51E-21	QMQIMDERKR	KRMLSNRESARRSRMRKQKHL	DDLMVQVSNV
BvbZIP4	7	3.51E-21	MDERKR	KRMASNRESARRSRMRKQKHL	DDLMAQADQL
BvbZIP6	194	4.02E-21	PLDKAAQQRQ	RRMIKNRESAARSRERKQAYQ	VELETLAMKL
BvbZIP24	235	4.60E-21	GNDPADVKRV	RRMLSNRESARRSRRRKQAHL	TELETQVSQL
BvbZIP41	197	1.27E-20	AVDRGTEKRL	KRKIKNRESAARSRARKQAYH	NELVSKVSQL
BvbZIP33	79	2.05E-20	PKNQVDERKH	RRMLSNRESARRSRMRKKRQI	NELWSHVLRL
BvbZIP32	82	2.05E-20	EASKPVEKVQ	RRLAQNREAARKSRLRKKAYI	QQLELGRSKL
BvbZIP28	26	2.91E-20	KYANMDERKR	KRMISNRESARRSRMKKQQHM	DEMLKEVNEL
BvbZIP48	149	3.26E-20	KGRTDDPKTL	RRLAQNREAARKSRLRKKAYV	QQLESSRLKL
BvbZIP39	209	3.26E-20	SDKVLDAKTL	RRLAQNREAARKSRLRKKAYV	QQLESSRIKL
BvbZIP20	167	3.26E-20	KEKVTDQKSL	RRLAQNREAARKSRLRKKAYV	QQLENSRLKL
BvbZIP19	290	3.26E-20	GPKTPDPKTL	RRLAQNREAARKSRLRKKAYV	QQLESSRIRL
BvbZIP12	173	3.26E-20	SKETKDQKTL	RRLAQNREAARKSRLRKKAYV	QQLESSRLKL
BvbZIP29	184	7.09E-20	SKTCIDERKR	KRMKSNRESAKRSRMRKQRHL	ENLRNNANEL
BvbZIP46	128	1.49E-19	DAELGGNPRH	KRMMKNRESAARSRARRQAYT	TQLEREHAEL
BvbZIP30	36	1.65E-19	PQQMMDLRKR	KRMESNRESARRSRIRKQKHM	DDLRAQTIEI
BvbZIP47	268	2.03E-19	VQDERELKRQ	KRKQSNRESARRSRLRKQAEC	EELQRRVESL
BvbZIP42	217	3.04E-19	QQLVVDPKRV	KRILANRQSAQRSRVRKLQYI	SELERSVTAL
BvbZIP27	306	3.04E-19	LQDEREIKRQ	RRKQSNRESARRSRLRKQAEC	DELAQRAEAL
BvbZIP8	81	3.04E-19	DAARLSEKVQ	RRLAQNREAAKKSRLRKKAYV	QQLESSRLKL
BvbZIP16	254	3.36E-19	VEDERELKRE	KRKQSNRESARRSRLRKQAEM	EELGKQVESL
BvbZIP2	283	4.10E-19	VQNEREIKRE	RRKQSNRESARRSRLRKQAET	EELARKVEAL
BvbZIP3	368	5.50E-19	EIAVSDPKRV	KRILANRQSAARSKERKLRYI	AELEHKVQTL
BvbZIP18	194	6.07E-19	ELALIDPKRA	KRIWANRQSAARSKERKMRYI	AELERKVQTL
BvbZIP11	181	6.07E-19	SDDPTDVKRM	RRMVSNRDSARRSRRRKQAHL	LQLEVEVEQL
BvbZIP38	189	1.18E-18	ELWTIDPKRA	KRILANRQSAARSKERKARYI	LELERKVQTL
BvbZIP40	97	1.30E-18	QSEEQEIRRL	KRMISNRESARRSRLRKRKQL	ENLQSQVLQL
BvbZIP5	204	2.49E-18	ELALIDPKRA	KRILANRQSAARSKERKIRYT	GELERRVQTL
BvbZIP13	112	2.99E-18	ELWIVDPKRA	KRILANRQSAARSKERKARYM	QELEKKVKSL
BvbZIP17	37	3.92E-18	GGFSSDEKKR	RRMESNRESARRSRQKKQQHL	DDLIREVSNL
BvbZIP45	416	5.60E-18	EIALTDPKRA	KRILANRLSAARSKERKMRYI	SELEHKVQTL
BvbZIP25	223	1.73E-17	DGTIDPKRVK	SRILANRQSAQRSRVRKLQYI	SELERSVTTL
BvbZIP14	95	4.72E-17	SPADKESKRL	KRLLRNRVSAQQARERKKAYL	SDLETRVKDL
BvbZIP7	146	8.34E-17	TDSDPLSKKR	KRQLRNRDAAMRSRERKKIYV	KDLEMKSRYM
BvbZIP23	92	3.44E-16	NPVDKEYRRL	KRLLRNRVSAQQARERKKVYV	NDLESRANEL
BvbZIP21	228	5.43E-16	GSNDGDDKRK	ARLMRNRESAQLSRQRKKQYV	EELEDKLRSM
BvbZIP36	179	1.66E-15	TEAEKEARRV	RRILANRESARQTIRRRQAYY	EELTRKAAEL
BvbZIP9	93	3.38E-14	NRCDTSSLKP	RKSLGNREAVRKYREKKKAHT	AYLEEEVKKL
BvbZIP44	234	3.76E-12	GSHAKTPSEN	DTKRAKQQFAQRSRVRKLQYI	AELERSVQSL
BvbZIP43	119	4.23E-12	SEELKSKKRG	RERGGNREAVRKYRQKKKAHA	ASLEDEVAKL
BvbZIP1	202	1.51E-11	SDSSHMKPPN	TDAKRKQHNARRSRVRKLQYI	AELERNAQAL

The above results indicate that the composition and distribution of conserved structural domains of sugar beet bZIP genes were similar in the same group but varied greatly among different groups. The similar composition and distribution patterns of exon-intron structures and conserved structural domains support the phylogeny and classification of the beet bZIP gene family.

### 3.3 Chromosomal location and collinearity analysis of the bZIP gene family in sugar beet

**FIGURE 4:**
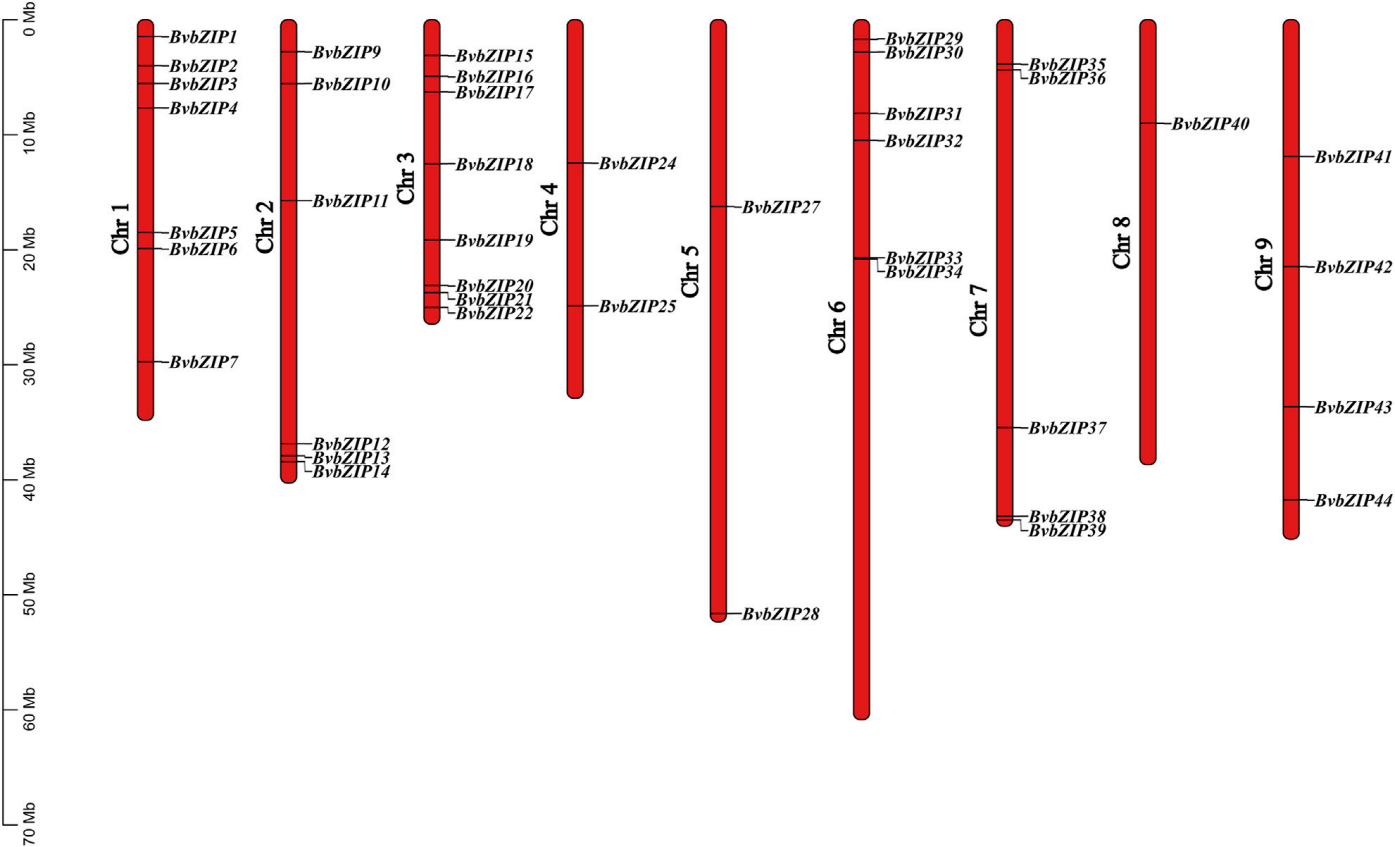
Locations of sugar beet bZIP genes on 9 chromosomes. The lengths of the chromosomes and the locations of the genes can be inferred from the scale on the left.

**FIGURE 5:**
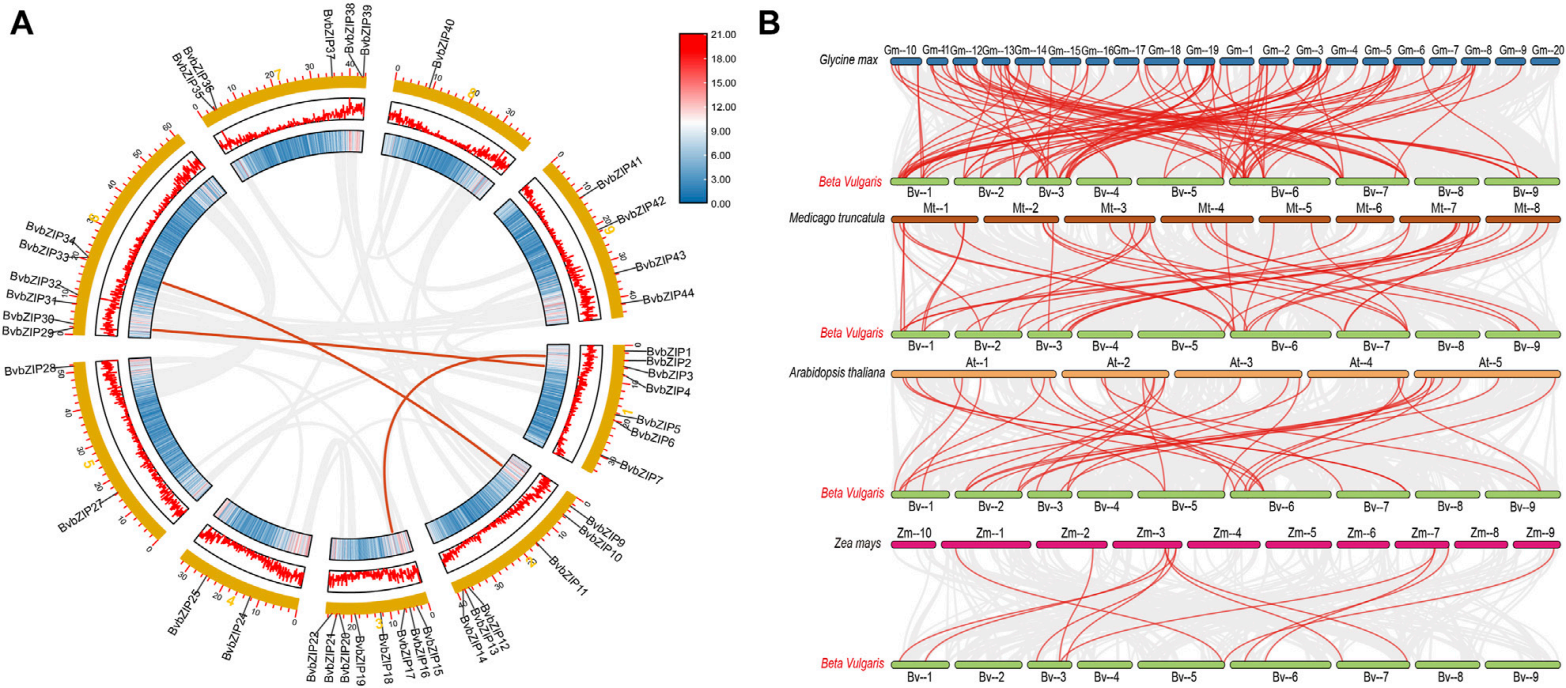
Collinearity analysis of the sugar beet bZIP gene family. **(A)** Chromosomes 1–9 are represented by yellow rectangles. The lines along the rectangles, heatmaps and histograms represent the density of genes on the chromosomes. Gray lines indicate cooccurrence blocks in the poplar genome, while red lines between chromosomes depict segmentally duplicated gene pairs; **(B)** Synteny analyses of the bZIPs between sugar beet and four representative plant species (*Glycine max*, *Arabidopsis thaliana*, *Medicago truncatula*, *Zea mays*). Gray lines on the background indicate collinear blocks of sugar beet and other plant genomes; red lines indicate sugar beet bZIP gene pairs.

The physical map showed that a total of 41 sugar beet bZIP genes were distributed in each of the 9 sugar beet chromosomes. From the graph it can be seen that Chr3 has the most bZIP genes with 8 genes while Chr8 has the least bZIP genes with the 1 gene. There are 6 genes distributed on chr6. The chromosomal positions of three bZIP genes (BvbZIP46–BvbZIP48) were not determined because they were on unanchored scaffolds (Figure 4). And although four genes (BvbZIP8, BvbZIP23, BvbZIP26, BvbZIP45) are identified on chromosomes, their specific locations are still uncertain. Gene duplication, such as tandem and segmental duplication, is important to large gene family evolution (Cannon et al., 2004). To understand the evolutionary mechanism of the bZIP gene family in sugar beet, we further analyzed tandem repeat events and fragment repeat events. We used the default parameters of the Advance Circos package in the TBtools to detect gene duplication (Chen et al., 2020). At the same time, we identified three gene pairs with fragment repeat events on sugar beet (Figure 5A). To further infer the phylogenetic relationship among the sugar beet bZIP genes, four syntenic maps were constructed for sugar beet and four other representative species (*A. thaliana*, *G. max*, *M. truncatula* and *Z. mays*) (Figure 5B). Among them, we found that *G. max* and sugar beet have the most duplicated gene pairs and *Z. mays* has the least.

### 3.4 Cis-acting elements of sugar beet bZIP gene family

**FIGURE 6:**
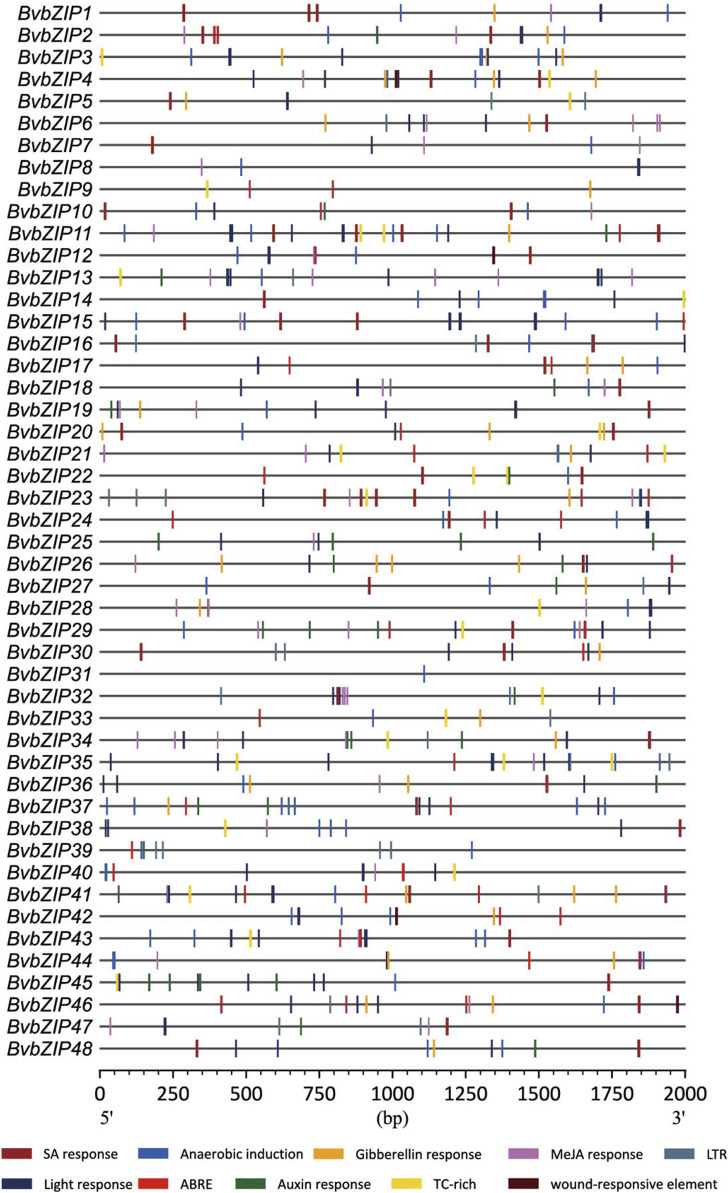
Cis-Acting element analysis of sugar beet bZIP gene promoters. ABRE, abscisic acid response; WUN-motif, wounding response; TC-rich, defense and stress responsive cis-acting element; SAresponse, salicylic acid response; Anaerobic induction, cis-acting element essential for the anaerobic induction; LTR, low-temperature response.

Cis-acting elements play a crucial role in the control of regulatory networks, including multi stimulus responsive genes, and determine that the stress-responsive expression pattern or tissue specificity of a gene is closely linked to the cis-elements in their promoter region (Abdullah et al., 2018). According to PlantCARE results, we identified multiple stress-responsive elements in the sugar beet bZIP family, and they were distributed among almost all members. Among them, 20 BvbZIPs contained defense and stress responsive elements (TC-rich), 40 contained anaerobic induction elements (Anaerobic induction), 18 contained low-temperature responsive elements (LTR), and 4 contained wound responsive elements (WUN-motif) (Figure 6).

In addition, all members of the BvbZIP family contain at least one cis-element associated with phytohormone response. For example, 30 sugar beet bZIP genes contain salicylic acid cis-elements (SA response), 34 sugar beet bZIP genes contain abscisic acid cis-elements (ABRE), 28 sugar beet bZIP genes contain jasmonic acid cis-elements (MeJA reponse), 20 sugar beet bZIP genes contain auxin reponse and 22 sugar beet bZIP genes contain gibberellin cis-elements (Gibberellin reponse). These cis-elements were visualized and analyzed by CFVisual software for cis-elements of the BvbZIP gene family (Figure 6) (Chen et al., 2022).

### 3.5 Predicted interacting protein networks

**FIGURE 7:**
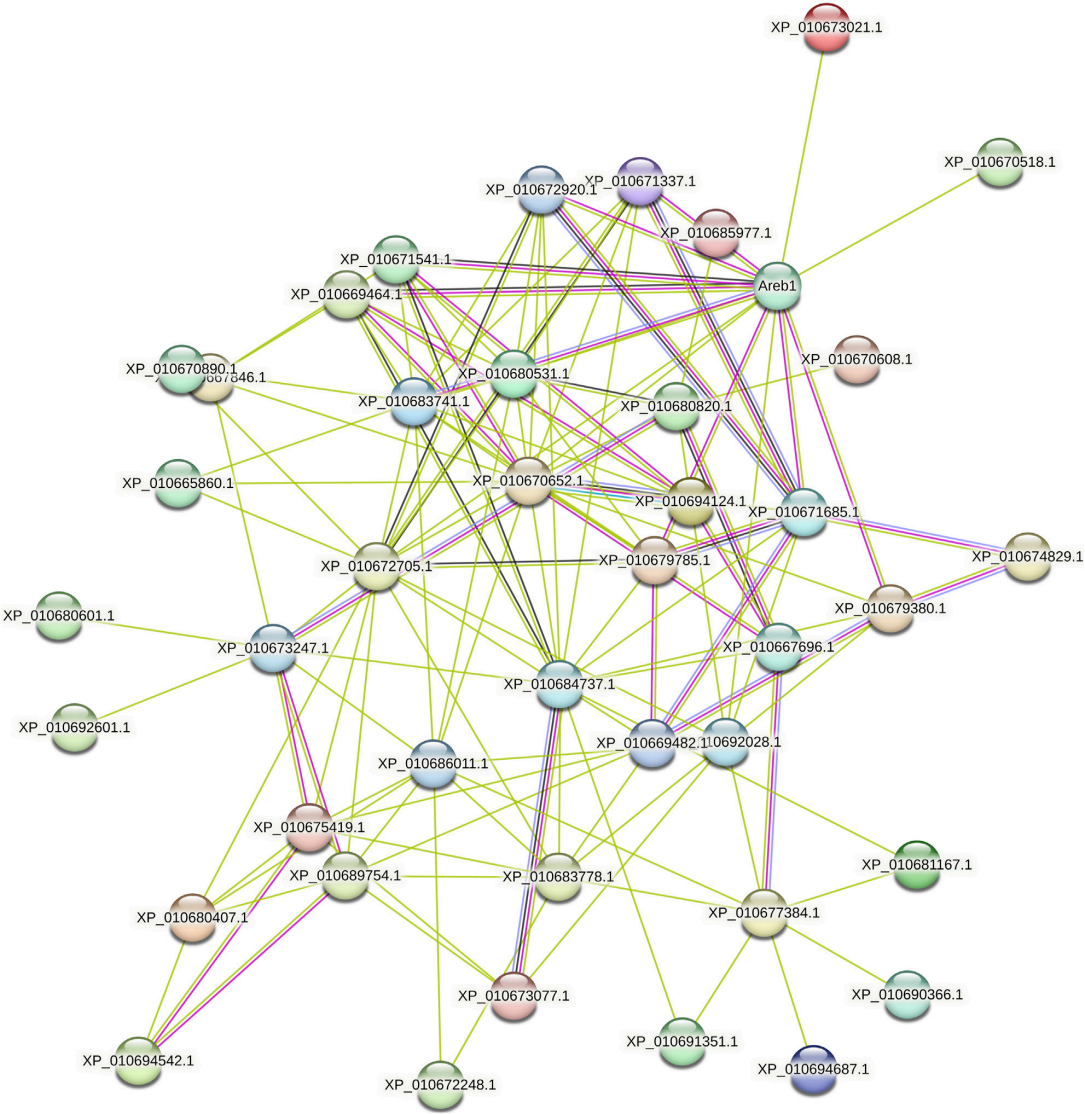
Interaction network of BvbZIP proteins.

Analysis of the interaction network of BvbZIP proteins and selection of key modules showed that a BvbZIP protein interacts closely with other proteins involved in light and ABA signaling responses. As shown in Figure 7, BvbZIPs have only one functional partner, Areb1. Interestingly, we found that XP_010671337.1 (BvbZIP15), XP_010672920.1 (BvbZIP4), XP_010671541.1 (BvbZIP16), XP_010669464.1 (BvbZIP2), XP_010683741.1 (BvbZIP35), XP_010679785.1 (BvbZIP30), XP_010671685.1 (BvbZIP17), XP_010679380.1 (BvbZIP28), XP_010673021.1 (BvbZIP20), XP_010670518.1 (BvbZIP12) and XP_010672705.1 (BvbZIP19) are associated with Areb1. The Areb1 binds to abscisic acid cis-elements response element (ABRE) in the promoter region of ABA-inducible genes. It plays an important role in stomatal closure by regulating ion fluxes in guard cells. Therefore, the BvbZIP gene may be related to the opening and closing of leaf stomatal in the disease resistance process of sugar beet.

### 3.6 BvbZIP genes indirectly mediate stomatal immunity

**FIGURE 8:**
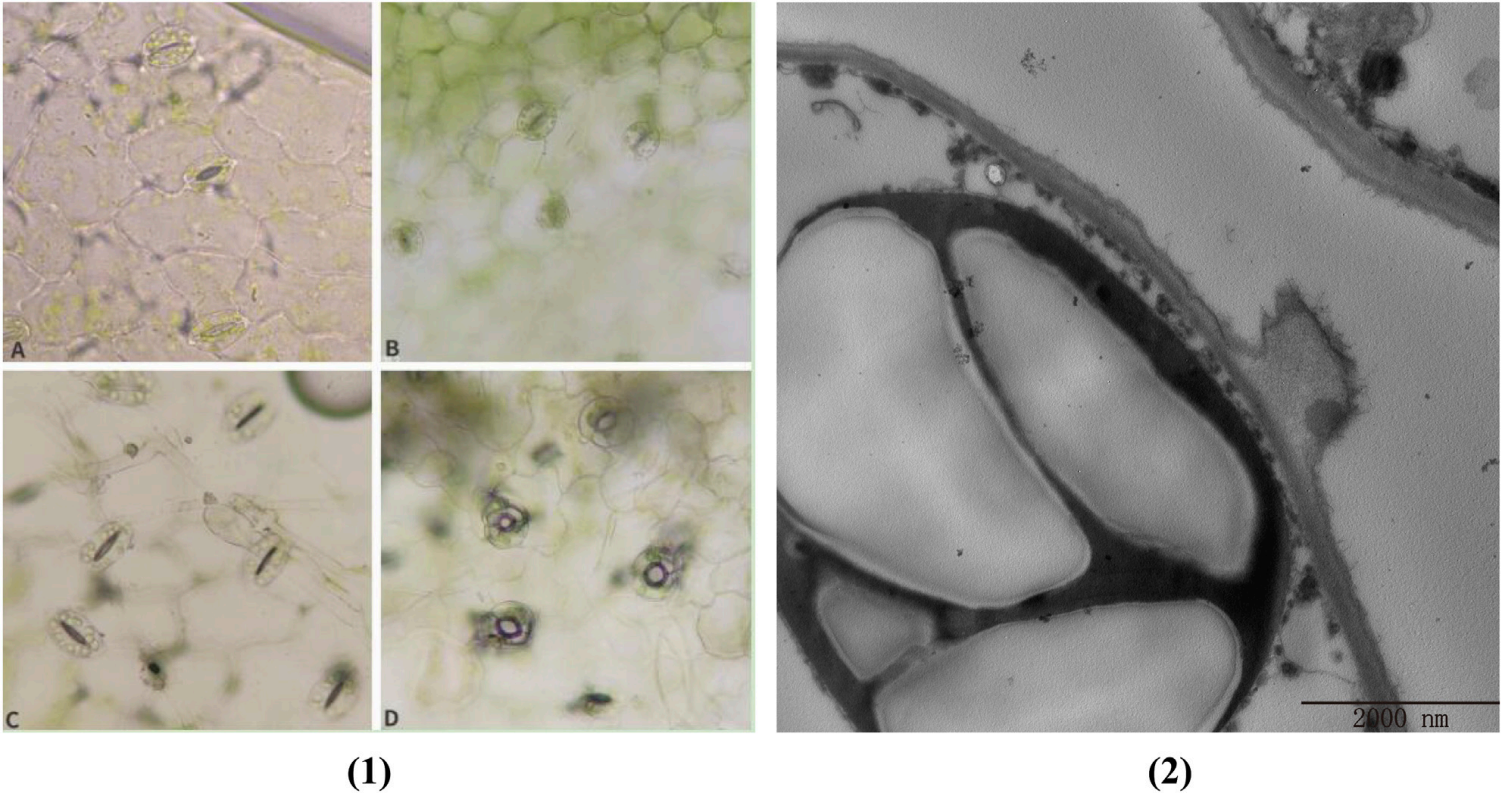
**(1)** Changes of leaf stomata of sugar beet **(A, B)** Normal leaf stomata; **(C)** Stomata of resistant varieties after 3 days of infection by *Cercospora beticola*; **(D)** Stomata of susceptible varieties after 3 days of infection by *C. beticola*); **(2)** Cell ultrastructure diagram.

Under natural growing conditions, plants have been exposed to a variety of potential pathogens and are constantly attacked by a variety of pathogens. In response to pathogen invasion, plants have evolved a complex immune system that utilizes innate physical and biochemical barriers to protect themselves from a variety of pathogens. Among them, stomata are the main way for many plant pathogens to enter plant tissues. Stomata consist of a pair of guard cells that are located in the natural openings of the plant. As the first barrier of plant defense against diseases, it determines whether the pathogen can penetrate the plant tissue for the next infection. Stomatal opening is also the main way for pathogens to infect plants. Plants have evolved a mechanism to regulate stomatal opening as an immune response against pathogen invasion. Plants fight against some pathogens infesting plants by regulating stomatal behavior (stomatal immunity) (Chen et al., 2019). As shown in Figure 8(1)A (F85621) and B (KWS5145), the stoma of normal sugar beet leaves are composed of two guard cells. The guard cells contain chloroplasts, and the stoma are unevenly distributed in the leaves. After 3 days of inoculation with *C. beticola*, the stomata of the leaves of the resistant varieties (F85621) were closed and were not infected, indicating that plants use the natural immune system to resist the invasion of *C. beticola*, however, it was found that the stomata of the leaves of the susceptible variety (KWS5145) were opened, the guard cells were destroyed, and the stomata could not be closed normally, indicating that *C. beticola* had destroyed the stomata of sugar beet leaves and prepared for the subsequent infection. (Figures 8C, D). Figure 8(2) is the ultrastructure of Figure 8C. From Figure 8(2), it can be clearly seen that the stomatal are immune to *C. beticola* after infection, so that they cannot infect the plant.

### 3.7 Observation of ultrastructure of sugar beet leaf cells

**FIGURE 9:**
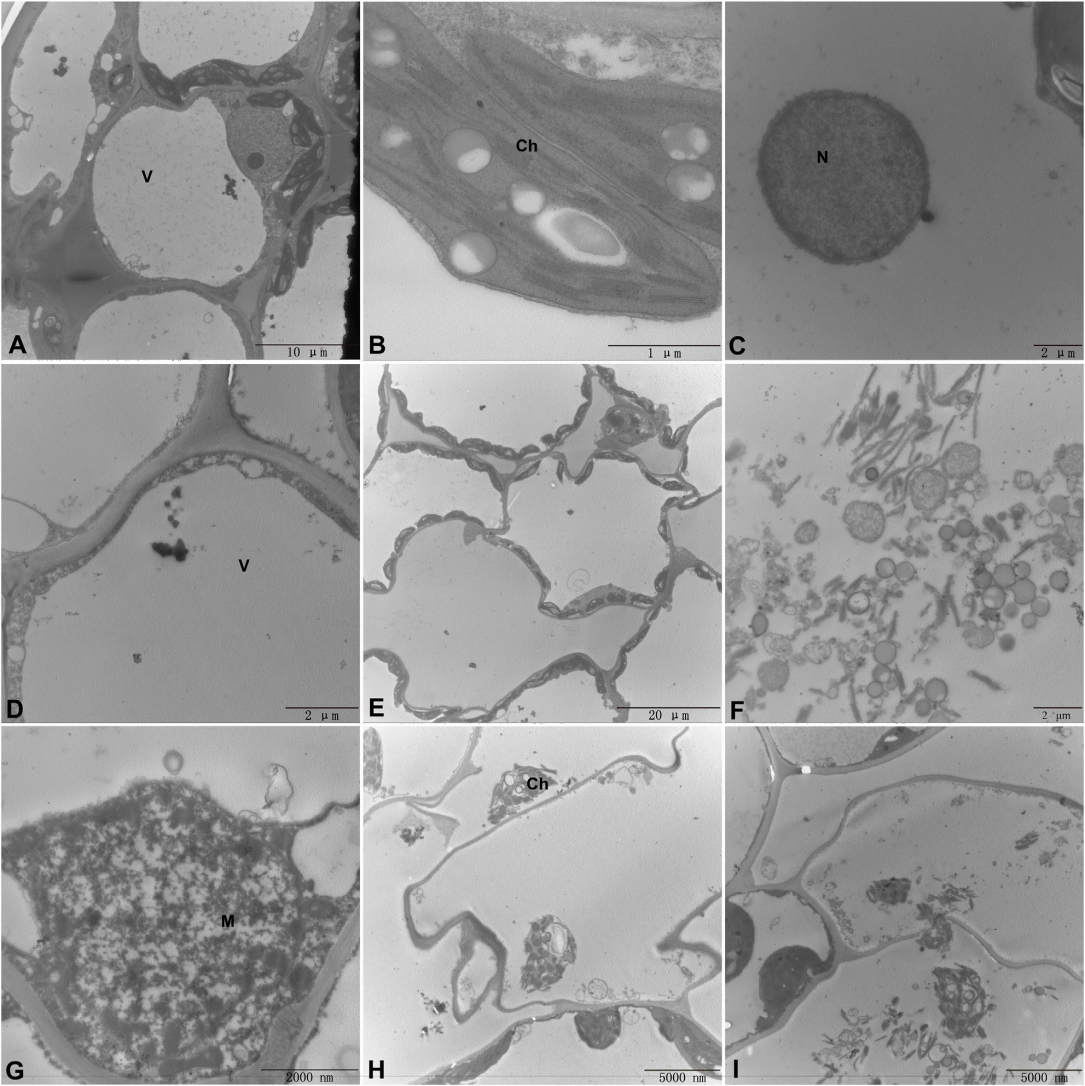
Ultrastructure of sugar beet leaf cells **(A–C)**. Normal leaf cell, chloroplast, mitochondrial structure **(D)**. Invagination of chloroplasts into the vesicles **(E)**. Organelle marginalization **(F)**. Leaf Chloroplast structure is completely destroyed **(G)**. Mitochondrial structure is disrupted **(H)**. Organelle structure is disrupted and deformed **(I)**. The cell wall is disrupted and organelles are exuded. (M, Mitochondria; Ch, Chloroplast; N, Nucleus; V, vacuole).

In order to study the effect of *C. beticola* on sugar beet leaf cells after infecting the internal tissue of sugar beet (KWS5145), the ultrastructure of sugar beet leaf cells was observed in this study. As shown in Figures 9A–C, the vacuole in normal cells occupies 2/3 of them. The nucleus, chloroplasts, mitochondria and other organelles are distributed around the vesicles (the mitochondria are intact; the chloroplasts are more regular oval; the structure of basal granule and thylakoid is clear). After the infection of *C. beticola*, the morphology of the cellular structure in sugar beet leaf cells started to undergo alterations. First, it can be observed that the vacuoles of the cells became obviously larger, and organelles such as chloroplasts and mitochondria began to marginalize (Figures 9D, E) then chloroplasts and mitochondria were destroyed, the double membrane structure was lost, the internal structure began to disintegrate, and the basal granule and thylakoid of chloroplasts were also destroyed (Figures 9F, G). Finally, the organelles in the cell were basically destroyed, the nucleus and cell wall were also decomposed, and the residual structure of the organelles began to seep out, and the whole cell died (Figures 9H, I). By observing the ultrastructure of sugar beet leaf cells, it can be seen that *C. beticola* has a strong destructive effect on sugar beet leaf cells. The destruction process of *C. beticola* began with the organelles inside the cells, and chloroplasts, mitochondria and vacuoles were first destroyed. Chloroplast and mitochondria are two important organelles in plant cells. Therefore, the destruction of the two organelles reduces the source of organic matter and energy production of the entire cell, which further reduces the resistance of the cell, and then destroys the overall structure of the cell, eventually leading to the death of the entire cell.

### 3.8 Expression analysis of BvbZIPs in highly resistant and susceptible varieties

**FIGURE 10:**
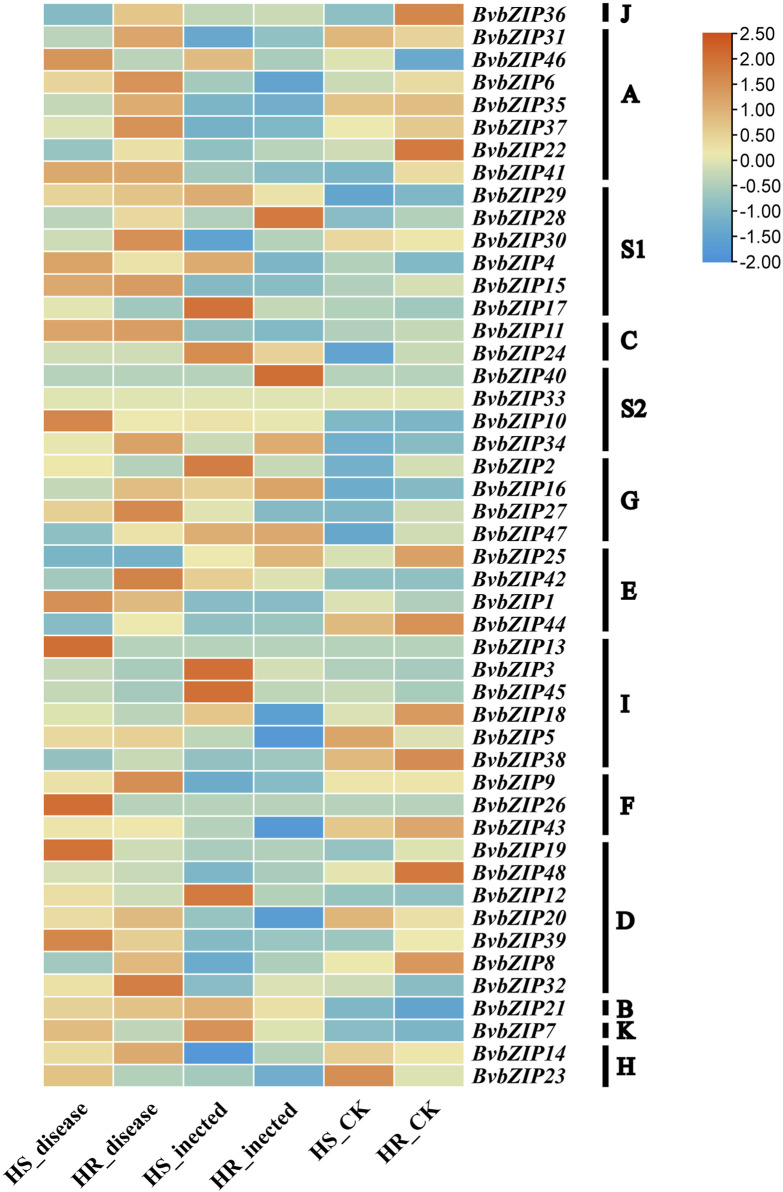
The expression patterns of BvbZIP gene family expression in highly resistant and highly susceptible varieties of sugar beet. (HS, refers to the highly susceptible variety; HR, refers to the highly resistant variety; inected, represents the infection of sugar beet CLS for 36 h; disease, represents the infection of sugar beet CLS for 96 h).

**FIGURE 11:**
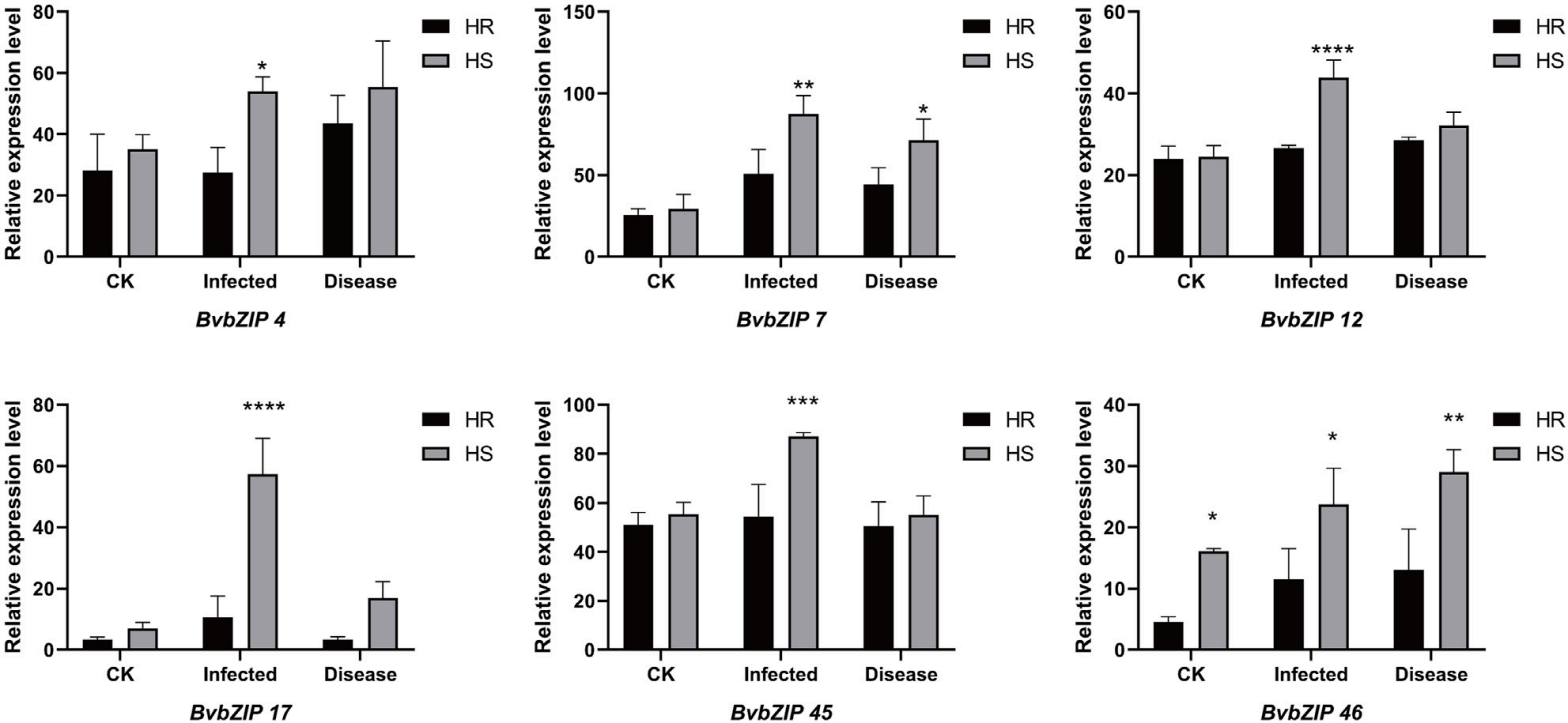
The expression patterns of selected BvbZIP genes in response to biotic stress treatments, Resistant varieties and susceptible varieties were compared. “HS” refers to highly susceptible variety; “HR” refers to highly resistant variety; “infected” represents the infection of sugar beet *C. beticola* for 36 h, “disease” represents the infection of sugar beet *C. beticola* for 96 h. Each treatment contained three biological replicates and the results obtained were expressed as means ± standard deviation. Duncan’s multiple range tests were utilized to determine the significance of differences *: *P < 0.05*; **: *P < 0.01*.

To analyze the potential role of the BvbZIPs in *C. beticola* leaf spot resistance, we performed *q*RT-PCR to identify the expression patterns of the BvbZIPs in high-susceptible and high-resistant varieties after 36 and 96 h of *C. beticola* infection (Figure 10). The relative expression levels are represented by fold change in Figure 11.The 44 BvbZIP genes, with the exception of BvbZIP13, BvbZIP26, BvbZIP33 and BvbZIP40, were expressed to various degrees in the leaf tissues. The expression of BvbZIP15 was the highest, and the expression of BvbZIP42 was the lowest. Six genes BvbZIP4, BvbZIP7, BvbZIP12, BvbZIP17, BvbZIP45 and BvbZIP46 were significantly upregulated after CLS infection. And the expression level of susceptible varieties is generally higher than that of resistant varieties. At the same time, it can be seen from Figure 10 that the expression of BvbZIP12, BvbZIP17 and BvbZIP45 was the most significant at 36 h after infection. Therefore, the differential expression of BvbZIP4, BvbZIP7, BvbZIP12, BvbZIP17, BvbZIP45 and BvbZIP46 could be related to the disease resistance of sugar beet.

### 3.9 Analysis of changes in SA and JA concentrations in sugar beet leaves under different conditions and validation of related genes by *q*RT-PCR

**FIGURE 12:**
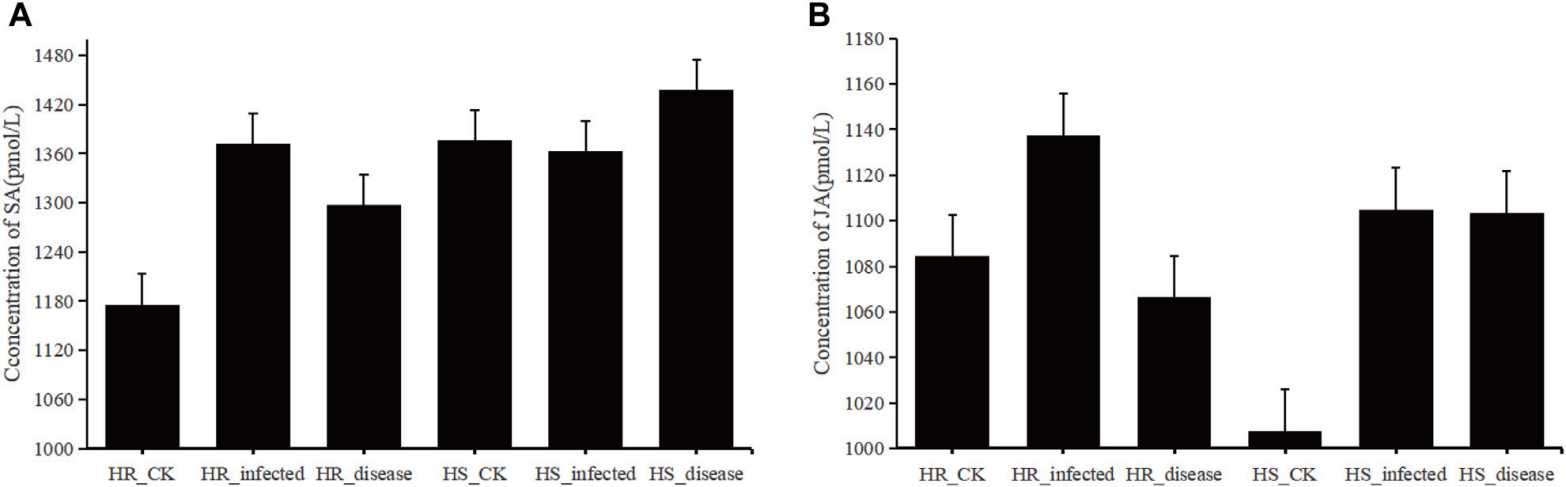
**(A)**, histogram of SA concentration in sugar beet leaves. **(B)**, histogram of JA concentration in sugar beet leaves.

SA concentration was measured in 6 groups of samples of highly resistant and highly susceptible varieties at the non-inoculated, early stage of *C. beticola*. (Figure 12A). In the highly resistant varieties, SA concentration increased significantly when *C. beticola*. invaded the sugar beet, and its increasing trend was the largest among all stages. SA concentration decreased from the beginning of infestation to the onset, but was still higher than in beet leaves that were not inoculated with the fungus. In susceptible varieties, SA concentration in beet leaves decreased after inoculation, but not significantly, and after entering the onset stage, SA concentration increased and was significantly higher than in the first two stages. In the highly resistant varieties, the JA concentrations in sugar beet leaves at the beginning of infestation were higher than in the control group (Figure 12B), while the JA concentrations were significantly reduced after entering the onset phase and were lower than the values of the control group. In the highly susceptible varieties, the JA concentrations at the beginning of infestation were much higher than those in the control group, while the concentrations at the onset were slightly lower than those at the beginning of the infestation.

**FIGURE 13:**
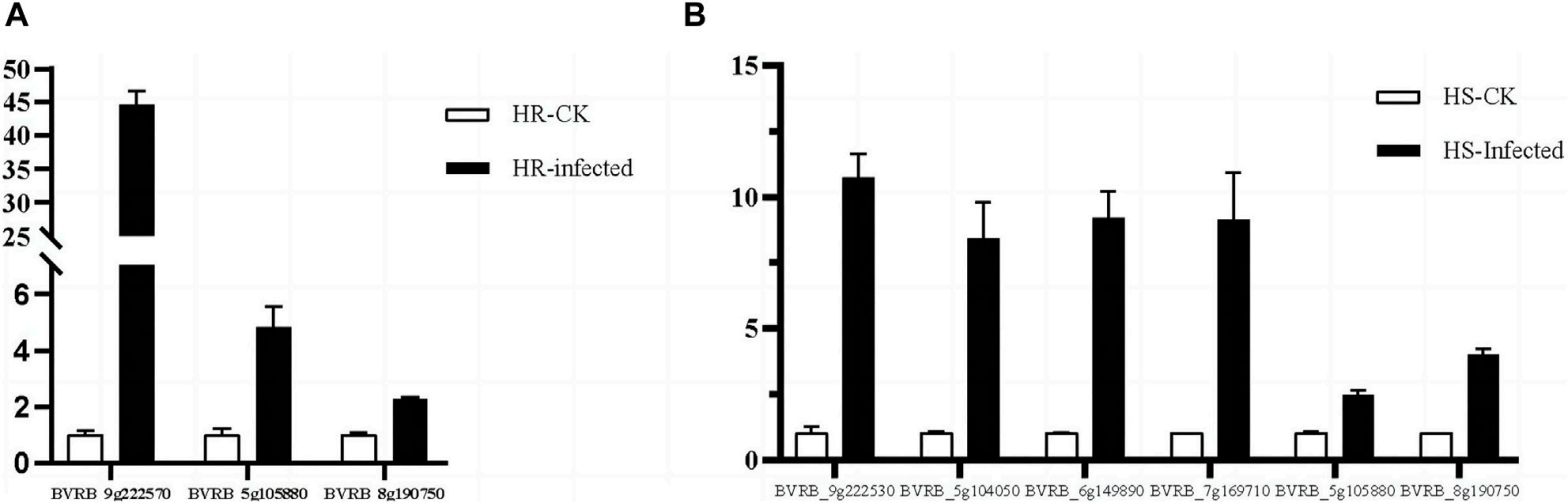
RT-qPCR analysis of signaling transduction genes. **(A)** genes associated with SA and JA signaling pathways at the beginning of infestation in highly resistant varieties. **(B)** genes associated with SA and JA signaling pathways at the beginning of infestation in highly susceptible varieties.

In the highly resistant and highly susceptible varieties, the genes related to SA and JA signal transduction pathways were verified by *q*RT-PCR at the beginning of infestation and in the control group (Figure 13). The *q*RT-PCR results were compared with the gene expression obtained from the transcriptome data (Majorbio.Co.limited) (Supplementary Figure S1), and it was found that the expression ratio of each gene in the early stage of infection was consistent with that of the control group.

## 4 Discussion

The bZIP gene family has been identified and comprehensively studied in many plants such as Arabidopsis, quinoa, maize, rice, cotton, potato, wheat, carrot, pomegranate etc. And the number of bZIP gene families varies with plant species. It is well known that bZIP proteins are mainly associated with abiotic and biotic stress responses in plants (Jakoby et al., 2002). Previous research on sugar beet was related to abiotic stress, such as salt stress (Gong et al., 2022). However, there was no research on the correlation between bZIP gene and biotic stress of sugar beet. In this study, a genome-wide characterization of the bZIP gene family in sugar beet was carried out using a bioinformatics approach. A total of 48 BvbZIP genes were identified and divided into A, B, C, D, E, F, G, H, I, J, K and S subfamily. Most evolutionary branches contain different numbers of genes in the two species, suggesting that they are closely related during evolution. However, there are exceptions, such as AtbZIP72, whose evolutionary branch does not contain any BvbZIP genes, suggesting that the role of this gene is more important in Arabidopsis than in sugar beet. In addition, the structure and function of the sugar beet bZIP gene family were predicted by analyzing the chromosome distribution and gene structure of BvbZIPs. BvbZIP genes are unevenly distributed on 9 chromosomes, and the positions of 4 BvbZIP genes on the chromosomes are still uncertain.

The infection process of the pathogen is that the pathogen contacts and invades the host plant, and expands and reproduces in the host plant. Finally, it leads to the disease of host plants and the process of obvious symptoms. At the same time, host plants also produce a series of responses to pathogen infection to defense, resistance, adaptive changes, and finally show symptoms. There are few reports on the infection process of *C. beticola*. CLS is an important foliar disease of Mungbean caused by *C. beticola* canescens, and its hyphae can be retained as endospores on plant leaves (Shahzady et al., 2017). Stoma are microscopic pores formed by pairs of guard cells in the epidermis of terrestrial plants; they are essential for gas exchange with the environment and controlling water loss (Zeng et al., 2010). At the same time, they are also the first barrier of plants against pathogens. During evolution, plants have evolved the ability to regulate stomatal not only in response to hormones such as ABA and various environmental factors such as light, air humidity and carbon dioxide, but also in response to pathogens (Gudesblat et al., 2009). Therefore, when the plant is infected with the disease, stoma can quickly close to prevent the pathogen from entering the internal tissue of the plant, effectively delaying the further invasion of the disease, so that the plant has more time to respond to the invasion of the disease, which is closely related to the disease resistance of the plant (Craenen et al., 1997). The ultrastructure of beet leaf cells was observed, Mycelium attaches to the surface of the leaf, closing the stomatal, which are disrupted and the mycelium enters the leaf cells. Therefore, the stomatal situation can be used as an indicator of whether the *C. beticola* has begun to infect. In addition, through the BvbZIPs protein interaction network, it can be found that the BvbZIP genes are related to Areb1 (Figure 7). And Areb1 is a basic domain/leucine zipper transcription factor that binds to the abscisic acid (ABA)-responsive element (ABRE) motif in the promoter region of ABA-inducible genes (Fujita et al., 2005). The plant hormone ABA regulates many important processes in plants, including seed germination and dormancy, stomatal closure, and adaptation to water stress (Finkelstein et al., 2002; Himmelbach et al., 2003). Under stress conditions, plants synthesize ABA in various organs and initiate defense mechanisms, such as the regulation of stoma aperture and expression of defense-related genes conferring resistance to environmental stresses. A typical effect of ABA on leaves is to reduce transpirational water loss by closing stomata and parallelly defend against microbes by restricting their entry through stomatal pores (Bharath et al., 2021). Thus, through the changes of stomata on the surface of sugar beet leaves, it can be inferred that the closure of stomata on the surface of sugar beet leaves may be related to the plant hormone ABA when the *C. beticola* infecting sugar beet leaves.

Organelles have essential functions in most cellular processes including growth and development of plants (Bittner et al., 2022). In this study, the destruction process of leaf cells by *C. beticola* was observed by observing the ultrastructure of sugar beet leaf cells (Figure 9). In the process of infection, *C. beticola* first destroys organelles of the cell, and the resistance of the entire cell will decrease after the organelles are destroyed. For example, chloroplast is a place for photosynthesis, which can synthesize organic matter needed for plant growth and development. The destruction of chloroplast will lead to the weakening of plant photosynthesis and affect the normal physiological activities of plants (Allen et al., 2011). Mitochondria are the place where plant cells produce energy (Horbay and Bilyy, 2016) Mitochondrial damage leads to a lack of energy supply to cells, which indirectly weakens plant resistance. This study will lay a foundation for further study on the pathogenic mechanism of CLS in the future.

Plant hormones are essential endogenous signaling molecules in plants. And it can regulate the growth and development of plants under severe stress conditions (Ryu and Cho, 2015). In this study, the concentrations of SA and JA in sugar beet leaves of high-resistant and high-susceptible varieties under different treatments were determined. It was found that the concentrations of SA and JA varied under different varieties and treatments (Figure 12). Based on the transcriptome data, the genes related to signal transduction pathway were screened from the differentially expressed genes in the early stage of infection of the two varieties compared with the control group, and their expression levels were statistically analyzed. The SA signal transduction pathway related gene *BVRB_9g222570*, *BVRB_5g105880* and *BVRB_8g190750* were screened in high resistant varieties to participate in the JA signal transduction pathway. Two genes related to SA signal transduction pathway and five genes related to JA signal transduction pathway were screened in high-susceptible varieties. *BVRB_9g222570* is a gene related to SA signal transduction pathway in high-resistant varieties. The ratio of its expression level to the control group at the initial stage of infection is 43.385, which is the highest among the selected genes, and it has specificity at the initial stage of infection of high-resistant varieties (Figure 13). It is speculated that it plays an important role in the process of SA signal transduction in the initial stage of high-resistant varieties against the infection of *C. beticola*. The protein interaction analysis of one gene related to SA signal transduction pathway and two genes related to JA signal transduction pathway in high-resistant varieties showed that the encoded proteins were identified as JAZ1, MYC2 and PR1, respectively (Supplementary Figure S2). The JAZ (Jasmonate Zim) domain protein has been identified as a JA signal inhibitor. The expression of JAZ1 is induced by JA and is also an auxin response gene (Grunewald et al., 2009). Therefore, it can be explained that there is a close molecular interaction between the JA signal transduction pathway and auxin in the highly resistant varieties of sugar beet in the early stage of resistance to *C. beticola* infection. MYC2 is a transcription factor that plays an important role in the JA signal transduction pathway. In response to the JA signal, the receptor COI1 induces the degradation of JAZ protein and releases downstream JA response genes, including MYC2 transcription factor (Chini et al., 2009; An et al., 2021). Thus one of the ways in which the 2 JA signaling pathway-related proteins interact is that JAZ protein degradation elicits a response from the MYC2 transcription factor. PR1 (Pathogensis Related 1) proteins are a series of proteins synthesized during defense against pathogen and are strongly associated with systemic acquisition of resistance (Han et al., 2023). This further validates that the SA signaling pathway is closely related to the systemic acquisition of resistance. By analyzing the cis-acting elements in the promoter regions of bZIP genes, we found that ABA-, SA-, MeJA-, and IAA-elements are present in the promoter regions of most bZIP genes, suggesting that these genes may be involved in hormone signaling pathways.

## 5 Conclusion

To date, bZIP transcription factors have been identified in different plant species and they are involved in a variety of key growth and physiological processes in plants. In this study, we identified 48 bZIP gene family members in the whole genome of sugar beet using a bioinformatics approach. We confirmed that BvbZIPs are involved in resistance to CLS. And in the process of observation and research on the stoma of leaves infested by *C. beticola*, it was found that *C. beticola* would first cover the stoma with mycelium in the process of infestation, and then destroy the stoma. The process of destruction of leaf cells by *C. beticola* was also observed in the ultrastructure of sugar beet leaf cells. In addition, we further demonstrated that the protein encoded by the SA signaling pathway-related gene *BVRB_9g222570* in high-resistant varieties is PR1, which is closely related to systemic acquired resistance. One of the protein interaction modes of JA signal transduction pathway is the response of MYC2 transcription factor caused by JAZ protein degradation, and there is a molecular interaction between JA signal transduction pathway and auxin. These findings may facilitate the further breeding of disease-resistance in sugar beet.

## Data Availability

The original contributions presented in the study are included in the article/Supplementary Material, further inquiries can be directed to the corresponding author.
