# Supplementary figures and images for: Genomic characterization of bZIP gene family and patterns of gene regulation on Cercospora beticola Sacc resistance in sugar beet (Beta vulgaris L.)
Source: Front Genet. 2024 Jul 30;15:1430589. doi: 10.3389/fgene.2024.1430589 (PMC11319121; doi:10.3389/fgene.2024.1430589)

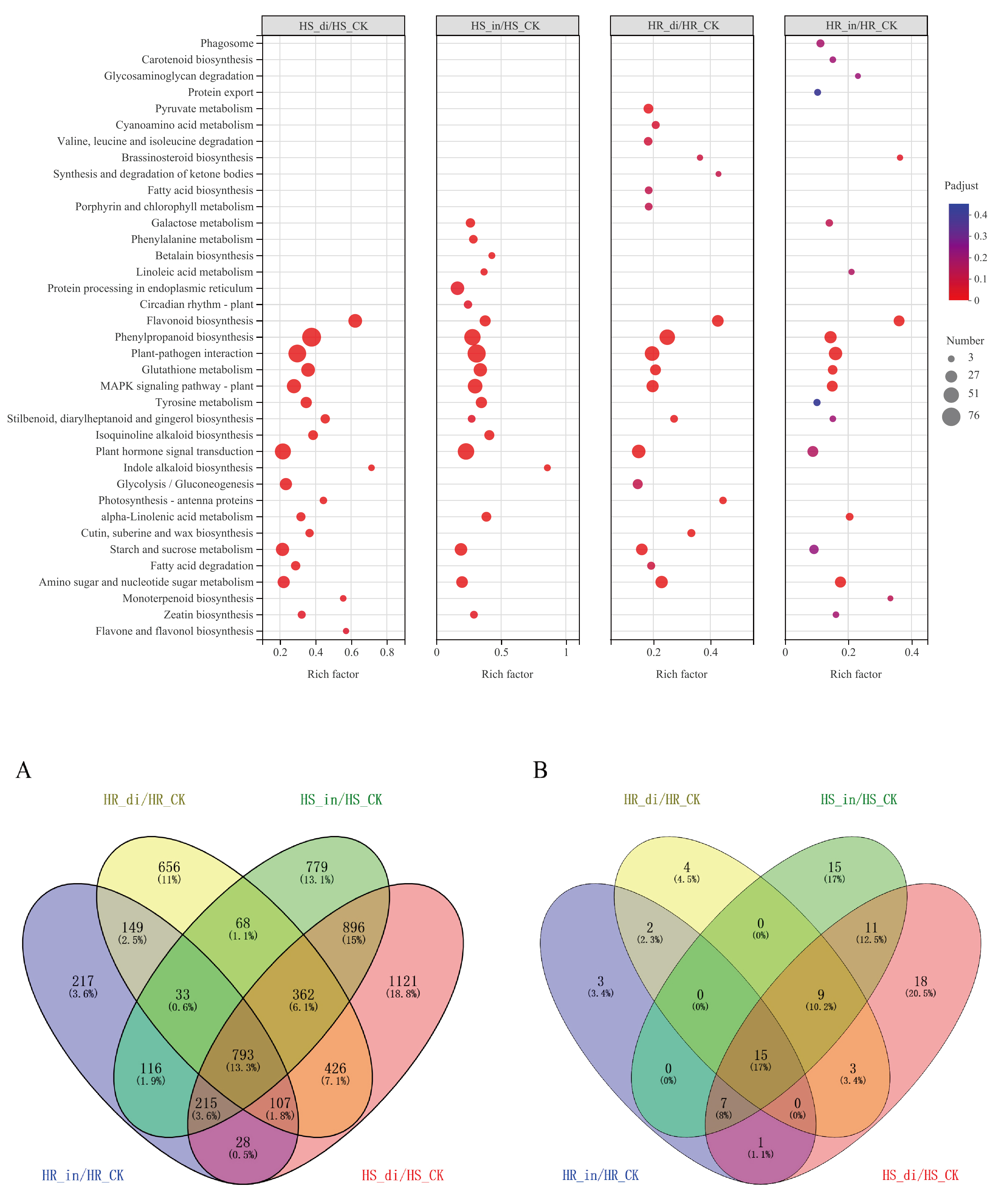

Supplement: Supplementary file 1 [file Image1.JPEG]

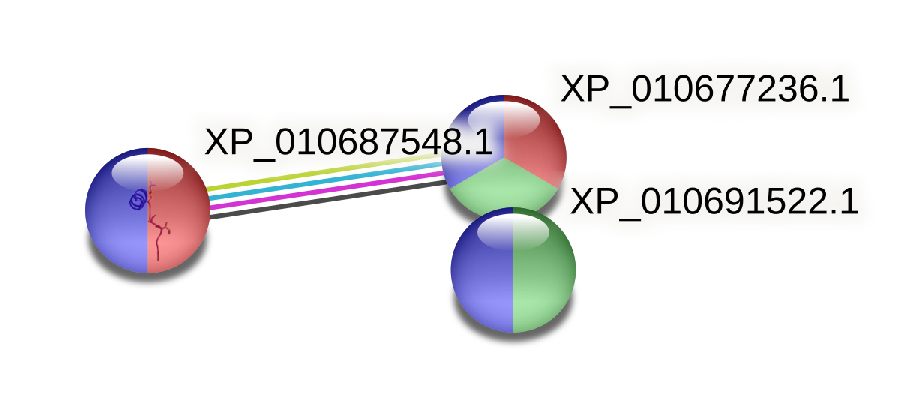

Supplement: Supplementary file 2 [file Image2.JPEG]
